# Transient analysis of the Erlang A model

**DOI:** 10.1007/s00186-015-0498-9

**Published:** 2015-08-21

**Authors:** Charles Knessl, Johan S. H. van Leeuwaarden

**Affiliations:** Department of Mathematics, Statistics and Computer Science, University of Illinois at Chicago, 815 South Morgan Street, Chicago, IL 60607-7045 USA; Department of Mathematics and Computer Science, Eindhoven University of Technology, P.O. Box 513, 5600 MB Eindhoven, The Netherlands

**Keywords:** Erlang A model, Queueing theory, Complex analysis, QED regime, Time-dependent analysis

## Abstract

We consider the Erlang A model, or $M/M/m+M$ queue, with Poisson arrivals, exponential service times, and *m* parallel servers, and the property that waiting customers abandon the queue after an exponential time. The queue length process is in this case a birth–death process, for which we obtain explicit expressions for the Laplace transforms of the time-dependent distribution and the first passage time. These two transient characteristics were generally presumed to be intractable. Solving for the Laplace transforms involves using Green’s functions and contour integrals related to hypergeometric functions. Our results are specialized to the $M/M/\infty$ queue, the *M* / *M* / *m* queue, and the *M* / *M* / *m* / *m* loss model. We also obtain some corresponding results for diffusion approximations to these models.

## Introduction

In many real-world systems customers that are waiting for service may decide to abandon the system before entering service. In the process of designing systems, it is important to understand the effect of this abandonment phenomenon on the system’s behavior. There has been a huge effort in developing models for systems that incorporate the effect of abandonments, also referred to as reneging or impatience (see, e.g., Dai et al. 2010; Garnett et al. 2002; Ward and Glynn 2005; Whitt 2004, 2006a, b; Kang and Ramanan 2010; Zeltyn and Mandelbaum 2004, 2005). The simplest yet widely used model is the completely Markovian $M/M/m+M$ model, also known as the Erlang A model. Its performance analysis has been an important subject of study in the literature (see for example Garnett et al. 2002; Whitt 2006c), not only because the Erlang A model is being used in practice (Mandelbaum and Zeltyn 2007), but also because it delivers valuable approximations for more general abandonment models (Whitt 2005).

The Erlang A model assumes Poisson arrivals with rate $\lambda$, exponential service times with mean $1/\mu$, *m* parallel servers, and most importantly, it incorporates the feature that waiting customers abandon the system after exponentially distributed times with mean $1/\eta$. Let *N*(*t*) denote the queue length at time *t*. Assuming independence across the interarrival, service and reneging times, the queue length process is a birth–death process $(N(t))_{t\ge 0}$. The stationary distribution of this process, and associated performance measures like delay or abandonment probabilities, are easy to obtain (Garnett et al. 2002; Mandelbaum and Zeltyn 2007). In contrast, studying the time-dependent behavior of $(N(t))_{t\ge 0}$ is generally judged to be prohibitively difficult (Fralix 2013; Ward 2012) because, among other things, the Kolmogorov forward equations do not seem to allow for a tractable solution. The main contributions of this paper are the exact solutions of both the forward and backward Kolmogorov equations, leading to exact expressions for the Laplace transforms of the time-dependent queue length distribution in Sect. 2 and first-passage times in Sect. 3.

The birth–death process describing the Erlang A model has birth rates, conditioned on $N(t)=j$, $\lambda _j=\lambda$ and death rates $\mu _j=j\mu$ for $j\le m$ and $\mu _j=m\mu +(j-m)\eta$ for $j>m$. There are available general results for the time-dependent behavior of birth–death processes. Karlin and McGregor (1957a, b, 1958) have shown that the backward and forward Kolmogorov equations satisfied by the transition probabilities of a birth–death process can be solved via the introduction of a system of orthogonal polynomials and a spectral measure. For each set of birth and death rates $(\lambda _j,\mu _j)$ there is an associated family of orthogonal polynomials. In some cases, when the set $(\lambda _j,\mu _j)$ is assumed to have a special structure, these orthogonal polynomials can be identified. One such special case is the *M* / *M* / *m* queue, with $\lambda _j=\lambda$ and $\mu _j=\min \{j,m\}\mu$. Notice that the Erlang A model incorporates the *M* / *M* / *m* queue as the special case $\eta \rightarrow 0^+$. Karlin and McGregor (1958) have shown for the *M* / *M* / *m* queue that the relevant orthogonal polynomials are the Poisson–Charlier polynomials. Determining the spectral measure, though, is rather complicated, which is why van Doorn (1979) made a separate study of determining the spectral properties of the *M* / *M* / *m* queue, starting from the general expression for the spectral measure in Karlin and McGregor (1958) in terms of the Stieltjes transform. For the same *M* / *M* / *m* queue, Saaty (1960) derived the Laplace transform of ${{\mathrm{\hbox {Prob}}}}[N(t)=n]$ over time, in terms of hypergeometric functions. As in Saaty (1960), we do not resort to the approach in Karlin and McGregor (1957a, b, 1958) for solving the Erlang A model, but instead opt to derive the explicit solution for the Laplace transform of ${{\mathrm{\hbox {Prob}}}}[N(t)=n]$ in a direct manner. The inverse transform then gives the desired solution for the time-dependent distribution, and we can also obtain the time-dependent moments. Mathematically, we shall use discrete Green’s functions, contour integrals, and special functions related to hypergeometric functions. Having explicit expressions for the Laplace transforms is useful for ultimately obtaining various asymptotic formulas, which would likely be simpler than the full solution and yield insight into model behavior.

Due to the cumbersome expressions for some of the stationary characteristics, and the presumed intractability of the time-dependent distribution, simpler analytically tractable processes $(D(t))_{t\ge 0}$ have been constructed that have similar time-dependent and stationary behaviors as $(N(t))_{t\ge 0}$. This can be done by imposing limiting regimes in which such approximating processes naturally arise as stochastic-process limits. Ward and Glynn (2002) make precise when the sample paths of the Erlang A model (and extensions using more general assumptions Ward and Glynn 2005) can be approximated by a diffusion process, where the type of diffusion process depends on the heavy-traffic regime. The diffusion process $(D(t))_{t\ge 0}$ is generally easier to study than the birth–death process $(N(t))_{t\ge 0}$, and can thus be employed to obtain simple approximations for both the stationary and the time-dependent system behavior. In Ward and Glynn (2002, 2003, 2005) the limiting diffusion process is a reflected Ornstein–Uhlenbeck process, whose properties are well understood (Fricker et al. 1999; Linetsky 2005; Ward and Glynn 2003). Garnett et al. (2002) proved a diffusion limit for the Erlang A model in another heavy-traffic regime, known as the Halfin–Whitt or QED regime. In this regime, the diffusion process $(D(t))_{t\ge 0}$ is a combination of two Ornstein–Uhlenbeck processes with different restraining forces, depending on whether the process is below or above zero. Both the stationary behavior (Garnett et al. 2002) and the time-dependent behavior (Leeuwaarden and Knessl 2012) of this process are well understood. From our general result for the Laplace transform of ${{\mathrm{\hbox {Prob}}}}[N(t)=n]$ we show how the results obtained in Leeuwaarden and Knessl (2012) for the above diffusion processes can be recovered. See the survey paper Ward (2012) for a comprehensive overview of diffusion approximations for many-server systems with abandonments.

The paper is structured as follows. In Sect. 2 we work towards Theorems 1 and 2 that provide explicit expressions for the Laplace transform of the time-dependent distribution of $(N(t))_{t\ge 0}$. In order to do so, we first reformulate the forward Kolmogorov equations in terms of difference equations in Sect. 2.1 and Laplace transforms in Sect. 2.2. In these steps we identify several key special functions of which some properties are proved in Sect.  2.3. Then, in Sect. 2.4 we prove Theorems 1 and 2 using all preliminary results in Sects. 2.1–2.3. In Sect. 2.5 we consider the limiting steady state case, and in Sect.  2.6 we treat the special cases $\eta =\mu =1$ ($M/M/\infty$ queue), $\eta \rightarrow 0^+$ (*M* / *M* / *m* queue) and $\eta \rightarrow \infty$ (the *M* / *M* / *m* / *m* loss model).

In Sect. 3 we follow a similar approach to obtain in Theorem 3 the Laplace transform of the distribution of the first time that $(N(t))_{t\ge 0}$ reaches some level $n_*>m$. This result is established in Sect. 3.1. We then again specialize the general results in Theorem 3 to several limiting cases. In particular, we derive in Sect. 3.2 results for the Halfin–Whitt regime. Finally, we give in Sect.  3.3 an expression for the mean first passage time.

## Transient distribution

### Expressions in terms of difference equations

We let *N*(*t*) be the number of customers in the system and set

2.1 $$\begin{aligned} p_n(t) ={{\mathrm{\hbox {Prob}}}}[N(t)=n\mid N(0)=n_0], \end{aligned}$$

so that $p_n(t)$ depends parametrically on the initial condition $n_0$, as well as the model parameters *m*, $\eta$ and $\rho =\lambda /\mu$. Since *N*(*t*) is a birth–death process with birth rate $\lambda$, death rate (setting $\mu =1$) *N*(*t*), for $N(t)\leqslant m$, and death rate $m+[N(t)-m]\eta$, for $N(t)\geqslant m$, the forward Kolmogorov equations are

2.2 $$\begin{aligned} p'_0(t)= & {} p_1(t)-\rho p_0(t)\end{aligned}$$

2.3 $$\begin{aligned} p'_n(t)= & {} \rho [p_{n-1}(t)-p_n(t)]+(n+1)p_{n+1}(t)-np_n(t),\quad 1\leqslant n\leqslant m-1,\end{aligned}$$

2.4 $$\begin{aligned} p'_m(t)= & {} \rho [p_{m-1}(t)-p_m(t)]+(m+\eta )p_{m+1}(t)-mp_m(t), \end{aligned}$$

and for $$n\geqslant m+1$$,

2.5 $$\begin{aligned} p'_n(t)=\rho [p_{n-1}(t)-p_n(t)]+[m+(n-m+1)\eta ]p_{n+1}(t) -[m+(n-m)\eta ]p_n(t)\nonumber \\ \end{aligned}$$

with the initial condition

2.6 $$\begin{aligned} p_n(0)=\delta (n,n_0), \end{aligned}$$

with $\delta (n,n_0)=1$ for $n=n_0$ and $\delta (n,n_0)=0$ for $n\ne n_0$. Setting

2.7 $$\begin{aligned} \widehat{P}_n(\theta )=\int ^{\infty }_0e^{-\theta t}p_n(t)\, dt \end{aligned}$$

and assuming that $0<n_0<m$ we obtain from (2.2) to (2.6)

2.8 $$\begin{aligned}&\displaystyle \widehat{P}_1(\theta )-(\rho +\theta )\widehat{P}_0(\theta )=0\end{aligned}$$

2.9 $$\begin{aligned}&\displaystyle (n+1)\widehat{P}_{n+1}(\theta )+\rho \widehat{P}_{n-1}(\theta )-(\rho +\theta +n)\widehat{P}_n(\theta )=-\delta (n,n_0),\quad 0< n<m,\end{aligned}$$

2.10 $$\begin{aligned}&\displaystyle [m+(n-m+1)\eta ]\widehat{P}_{n+1}(\theta ) +\rho \widehat{P}_{n-1}(\theta )\nonumber \\&\displaystyle -[\rho +\theta +m+(n-m)\eta ]\widehat{P}_n(\theta )=0, \quad n\geqslant m. \end{aligned}$$

If $n_0=0$ the right side of (2.8) must be replaced by $-1$, while if $n_0\geqslant m$ the right side of (2.10) must be replaced by $-\delta (n,n_0)$, and then the right side of (2.9) is zero. We proceed to explicitly solve (2.8)–(2.10), distinguishing the cases $0<n_0<m$ and $n_0>m$, and then we show that the results also apply for $n_0=m$ and $n_0=0$.

### Special function solutions to the difference equations

Since the coefficients in the difference equations in (2.9) and (2.10) are linear functions of *n*, we can solve these explicitly with the help of contour integrals. First consider the integral

2.11 $$\begin{aligned} F_n(\theta )\equiv \dfrac{1}{2\pi i}\int _{C_0}\dfrac{e^{\rho z}}{z^{n+1}(1-z)^{\theta }}\ dz, \end{aligned}$$

where $C_0$ is a small circle in the *z*-plane, on which $|z|<1$. The integrand in (2.11) is analytic inside the unit circle, if we define

$$\begin{aligned} (1-z)^{\theta }=|1-z|^{\theta }e^{i\theta \arg (1-z)} \end{aligned}$$

with $|\arg (1-z)|<\pi$, so that for *z* real and $z<1$, $\arg (1-z)=0$. By expanding

2.12 $$\begin{aligned} (1-z)^{-\theta }=1+\theta z+\theta (\theta +1)z^2/2!+\dots \end{aligned}$$

as a binomial series, we obtain the alternate form

2.13 $$\begin{aligned} F_n(\theta )&=\sum ^n_{\ell =0}\dfrac{\rho ^{n-\ell }}{(n-\ell )!}\dfrac{\theta (\theta +1)\dots (\theta +\ell -1)}{\ell !}\nonumber \\&=\sum ^n_{\ell =0}\dfrac{\rho ^{n-\ell }}{(n-\ell )!\ell !}\dfrac{\Gamma (\theta +\ell )}{\Gamma (\theta )}, \end{aligned}$$

where $\Gamma (\cdot )$ is the Gamma function. It follows that $F_{-1}(\theta )=0$, $F_0(\theta )=1$ and $F_1(\theta )=\rho +\theta$, and hence $F_n(\theta )$ satisfies Eq. (2.8). Furthermore, from (2.11) we have

2.14 $$\begin{aligned} \rho (F_{n-1}&-F_n) +(n+1)F_{n+1}-(n+\theta )F_n\nonumber \\&=\dfrac{1}{2\pi i}\int _{C_0}\dfrac{e^{\rho z}}{z^{n+1}}\dfrac{1}{(1-z)^{\theta }}\left[ \rho (z-1)+\dfrac{n+1}{z}-n-\theta \right] \, dz\nonumber \\&=-\dfrac{1}{2\pi i}\int _{C_0}\, \dfrac{d}{dz}\left[ \dfrac{e^{\rho z}}{z^{n+1}(1-z)^{\theta -1}}\right] \, dz=0, \end{aligned}$$

as the contour $C_0$ is closed and the integrand in (2.14) is a perfect derivative. Thus $F_n(\theta )$ provides a solution to the homogeneous version of (2.9) (with the right side replaced by zero). We shall solve (2.8)–(2.10) using a discrete Green’s function approach, and this will require a second, linearly independent, solution to (2.9). Such a solution may be obtained by using the same integrand as in (2.11) but integrating over a different contour. Thus we let

**Fig. 1:**
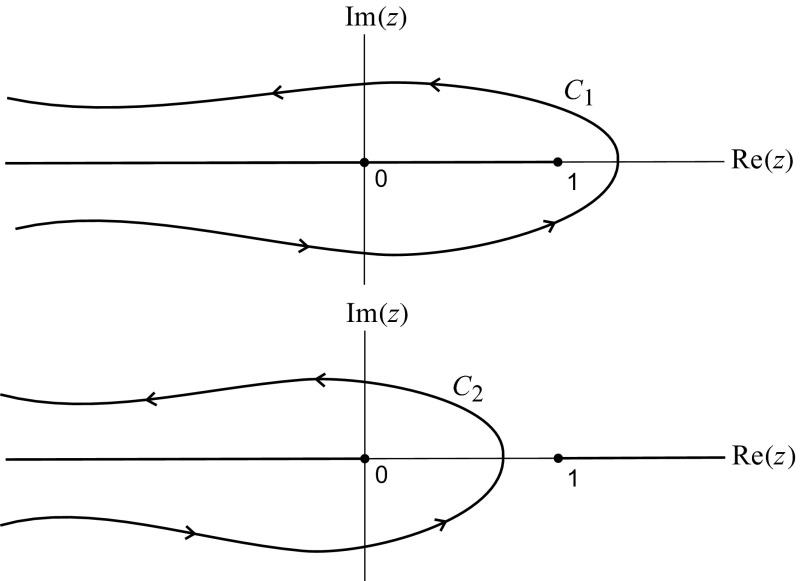
A sketch of the branch cuts and the contours $C_1$ and $C_2$

2.15 $$\begin{aligned} G_n(\theta )=\dfrac{1}{2\pi i}\int _{C_1}\dfrac{e^{\rho z}}{z^{n+1}(z-1)^{\theta }}\, dz, \end{aligned}$$

where $$C_1$$ goes from $$-\infty -i\varepsilon $$ to $$-\infty +i\varepsilon $$$$(\varepsilon >0)$$, encircling $$z=1$$ in the counterclockwise sense (see Fig. 1). In (2.15) we use the branch $$(z-1)^{\theta }=|z-1|^{\theta }e^{i\theta \arg (z-1)}$$, where $$|\arg (z-1)|<\pi $$, so the integrand is analytic in $$\mathbb {C}-\{{{\mathrm{\hbox {Im}}}}(z)=0, {{\mathrm{\hbox {Re}}}}(z)\leqslant 1\}$$. By a calculation completely analogous to (2.14), and noting that $$C_1$$ begins and ends at $$z=-\infty $$, where the integrand in (2.15) decays exponentially to zero, we see that $$G_n(\theta )$$ satisfies the homogeneous form of (2.9). However, $$G_n$$ does not satisfy the boundary equation in (2.8), and we now have

2.16 $$\begin{aligned} G_{-1}(\theta )=\dfrac{1}{2\pi i}\int _{C_1}\dfrac{e^{\rho z}}{(z-1)^{\theta }}\, dz=\dfrac{e^{\rho }\rho ^{\theta -1}}{\Gamma (\theta )}. \end{aligned}$$

Now consider (2.10). We shall again construct two independent solutions to this difference equation. Let $f_n$ satisfy $[\rho +\theta +m+(n-m)\eta ]f_n=\rho f_{n-1}+[m+(n-m+1)\eta ]f_{n+1}$ and represent $f_n$ as a contour integral, with

2.17 $$\begin{aligned} f_n=\int _C z^{-n-1}\mathcal {F}(z)\, dz, \end{aligned}$$

for some function $\mathcal {F}(\cdot )$ and contour *C*. Then we have

2.18 $$\begin{aligned} \int _C\dfrac{1}{z^{n+1}}\bigg [\rho +\theta +m+(n-m)\eta -\rho z -\dfrac{m}{z}-\dfrac{(n-m+1)\eta }{z}\bigg ]\mathcal {F}(z)\, dz=0.\nonumber \\ \end{aligned}$$

We use integration by parts in (2.18) with

2.19 $$\begin{aligned} \int _C\dfrac{n}{z^{n+1}}\mathcal {F}(z)\, dz= \int _C\dfrac{z\mathcal {F}'(z)}{z^{n+1}}\, dz \end{aligned}$$

and for now assume that *C* is such that there are no boundary contributions arising in (2.19), from endpoints of *C*. Using (2.19) in (2.18) we can rewrite (2.18) as a contour integral of $z^{-n-1}$ times a function of *z* only, and if (2.18) is to hold for all *n* we argue that this function must vanish. We thus obtain the following differential equation for $\mathcal {F}(z)$:

2.20 $$\begin{aligned} \eta \mathcal {F}'(z)(z-1)+\mathcal {F}(z)\bigg [\rho +\theta +m(1-\eta )-\rho z -\dfrac{m}{z}(1-\eta )\bigg ]=0, \end{aligned}$$

whose solution is, up to a multiplicative constant,

2.21 $$\begin{aligned} \mathcal {F}(z)=e^{\rho z/\eta }(z-1)^{-\theta /\eta }z^mz^{-m/\eta }. \end{aligned}$$

Now we use (2.21) in (2.17) and make two different choices of *C* and different branches of (2.21), to obtain two independent solutions to (2.10). Note that now (2.21) has branch points both at $z=1$ and $z=0$. We let

2.22 $$\begin{aligned} H_n(\theta ; m)= \dfrac{1}{2\pi i} \int _{C_1} \dfrac{e^{\rho z/\eta }}{(z-1)^{\theta /\eta }z^{n+1-m}z^{m/\eta }}\, dz, \end{aligned}$$

where $C_1$ is as in (2.15) (or Fig. 1) and the branch of $(z-1)^{\theta /\eta }$ is

2.23 $$\begin{aligned} |z-1|^{\theta /\eta }\exp [i\theta \arg (z-1)/\eta ] \end{aligned}$$

with $|\arg (z-1)|<\pi$, and $z^{m/\eta }=|z|^{m/\eta }\exp [im(\arg z)/\eta ]$ with $|\arg (z)|<\pi$.

Then the integrand in (2.22) is analytic in $\mathbb {C}- \{{{\mathrm{\hbox {Im}}}}(z)=0, {{\mathrm{\hbox {Re}}}}(z)\leqslant 1\}$. For the second solution we set

2.24 $$\begin{aligned} I_n(\theta ; m)=\dfrac{1}{2\pi i}\int _{C_2}\dfrac{e^{\rho z/\eta }}{(1-z)^{\theta /\eta }z^{n+1-m}z^{m/\eta }}\, dz, \end{aligned}$$

where $C_2$ goes from $-\infty -i\varepsilon$ to $-\infty +i\varepsilon$, encircling $z=0$ in the counterclockwise sense, and $(1-z)^{\theta /\eta }$ is defined to be analytic exterior to the branch cut where ${{\mathrm{\hbox {Im}}}}(z)=0$ and ${{\mathrm{\hbox {Re}}}}(z)\geqslant 1$, similarly to (2.11). Also, $z^{m/\eta }$ is defined as below (2.22), so the integrand in (2.24) is analytic exterior to the branch cuts where ${{\mathrm{\hbox {Im}}}}(z)=0$ and ${{\mathrm{\hbox {Re}}}}(z)\leqslant 0$ or ${{\mathrm{\hbox {Re}}}}(z)\geqslant 1$, and in particular on the contour $C_2$ (see again Fig. 1).

We have thus shown that the general solution to (2.10) is a linear combination of $H_n$ and $I_n$, while that of (the homogeneous version of) (2.9) is a combination of $F_n$ and $G_n$.

### Properties of the key special functions

We now establish several useful properties of these functions. The integrals in (2.11), (2.15), (2.22) and (2.24) may all be expressed in terms of generalized hypergeometric functions, but we shall not use this fact. First, we note that if $\eta =1$ then $F_n=I_n$ and $G_n=H_n$. The latter is obvious since (2.15) and (2.22) have the same contour $C_1$, while if $\eta =1$ in (2.24) the branch point at $z=0$ disappears and $C_2$ may be deformed to the loop $C_0$ in (2.11).

The functions $H_n$ and $I_n$ have very different asymptotic behaviors as $n\rightarrow \infty$. For *n* large, standard singularity analysis shows that the asymptotics of $I_n$ are governed by the singularity at $z=1$, and then setting $z=1-\xi /n$ and letting $n\rightarrow \infty$ in (2.24) yields

2.25 $$\begin{aligned} I_n&\sim e^{\rho /\eta }\dfrac{1}{2\pi i}\int _{ C_{\xi }}n^{\theta /\eta -1}e^{\xi }\xi ^{-\theta /\eta }\, d\xi =n^{\theta /\eta -1}\dfrac{e^{\rho /\eta }}{\Gamma (\theta /\eta )},\quad n\rightarrow \infty . \end{aligned}$$

Here $C_{\xi }$ goes from $-\infty -i\varepsilon$ to $-\infty +i\varepsilon$, with $\varepsilon >0$, and encircles $\xi =0$. Thus $I_n$ has an algebraic dependence on *n* for *n* large. To expand $H_n$ we can simply dilate the contour $C_1$ in (2.22) to the range $|z|\gg 1$ and then expand $(z-1)^{\theta /\eta }=z^{\theta /\eta }[1-\theta /(\eta z) +O(z^{-2})]$. We thus obtain

2.26 $$\begin{aligned} H_n(\theta )&\sim \left( \dfrac{\rho }{\eta }\right) ^{n-m+\frac{\theta +m}{\eta }}\dfrac{1}{\Gamma \left( n+1+\frac{m+\theta }{\eta }-m\right) }\nonumber \\&\sim \dfrac{1}{n!}\left( \dfrac{\rho }{\eta }\right) ^{n-m+\frac{\theta +m}{\eta }}n^{m-\frac{m+\theta }{\eta }},\quad n\rightarrow \infty , \end{aligned}$$

and hence $H_n$ decays roughly as 1 / *n*!. Here we also used $\Gamma (n+x)\sim \Gamma (n)n^x$, which holds for $n\rightarrow \infty$ and *x* fixed. Next we consider the discrete Wronskian

2.27 $$\begin{aligned} W_n=W_n(\theta ;m)=H_n(\theta ;m)I_{n+1}(\theta ;m)-H_{n+1}(\theta ;m)I_n(\theta ;m). \end{aligned}$$

Using the fact that $H_n$ and $I_n$ satisfy (2.10) we find that

2.28 $$\begin{aligned} \rho (I_nH_{n-1}-I_{n-1}H_n) +[m+(n-m+1)\eta ][I_nH_{n+1}-H_nI_{n+1}]=0 \end{aligned}$$

and thus $W_n[m+(n-m+1)\eta ]=\rho W_{n-1}$. Solving this simple recurrence leads to

2.29 $$\begin{aligned} W_n(\theta ;m)=\omega _*(\theta ;m)\left( \dfrac{\rho }{\eta }\right) ^n\dfrac{1}{\Gamma \left( n+\frac{m}{\eta }-m+2\right) }. \end{aligned}$$

To determine $\omega _*(\cdot )$ we let $n\rightarrow \infty$ in (2.27), and use (2.25) and (2.26). Then $H_{n+1}\ll H_n$ with $I_{n+1}=O(I_n)$, so that

2.30 $$\begin{aligned} W_n\sim H_nI_{n+1}\sim \dfrac{1}{n!}\dfrac{e^{\rho /\eta }}{\Gamma \left( \frac{\theta }{\eta }\right) }\left( \dfrac{\rho }{\eta }\right) ^{n-m+\frac{\theta +m}{\eta }}n^{m-1-\frac{m}{\eta }},\quad n\rightarrow \infty . \end{aligned}$$

Comparing (2.29) with (2.30) we determine $\omega _*(\cdot )$, and thus

2.31 $$\begin{aligned} W_n=\dfrac{e^{\rho /\eta }}{\Gamma \left( \frac{\theta }{\eta }\right) }\left( \dfrac{\rho }{\eta }\right) ^{n-m+\frac{\theta +m}{\eta }}\dfrac{1}{\Gamma \left( n-m+2+\frac{m}{\eta }\right) }. \end{aligned}$$

A completely analogous calculation shows that

2.32 $$\begin{aligned} \widetilde{W}_n=-G_{n+1}F_n+G_nF_{n+1}=\dfrac{e^{\rho }}{\Gamma (\theta )}\dfrac{\rho ^{n+\theta }}{(n+1)!}, \end{aligned}$$

which also follows by setting $\eta =1$ in (2.31).

### Main results: solutions of the difference equations

We solve (2.8)–(2.10) for $0<n_0<m$, writing the solution as

2.33 $$\begin{aligned} \widehat{P}_n(\theta )={\left\{ \begin{array}{ll} A F_n(\theta ), &{}0\leqslant n \leqslant n_0\\ B F_n(\theta )+C G_n(\theta ),&{}n_0\leqslant n\leqslant m\\ D H_n(\theta ;m),&{}n\geqslant m. \end{array}\right. } \end{aligned}$$

Then $\widehat{P}_n$ will decay faster than exponentially as $n\rightarrow \infty$, in view of (2.26), and satisfy the boundary equation in (2.8), since $F_n$ does but $G_n$ does not. It remains only to determine *A*, *B*, *C*, *D*; these functions will depend only on $\theta$ and the parameters *m*, $\eta$, $\rho$. By continuity at $n=n_0$ and $n=m$, we have

2.34 $$\begin{aligned} \begin{aligned}&A F_{n_0}=B F_{n_0}+C G_{n_0}\\&B F_m+C G_m=D H_m. \end{aligned} \end{aligned}$$

Then using (2.10) with $n=m$ leads to

2.35 $$\begin{aligned} (m+\theta +\rho )D H_m=\rho (B F_{m-1}+C G_{m-1})+(m+n)D H_{m+1}, \end{aligned}$$

and using (2.9) with $n=n_0$ (then $\delta (n,n_0)=1$) leads to

2.36 $$\begin{aligned} \rho A F_{n_0-1}+(n_0+1)(B F_{n_0+1}+D G_{n_0+1}) -(\rho +\theta +n_0)A F_{n_0}=-1. \end{aligned}$$

If we introduce *a* and $\alpha$ by setting

2.37 $$\begin{aligned} A= & {} a[F_{n_0}+\alpha G_{n_0}]H_m\end{aligned}$$

2.38 $$\begin{aligned} B= & {} aF_{n_0}H_m,\quad C=\alpha aF_{n_0}H_m\end{aligned}$$

2.39 $$\begin{aligned} D= & {} a[F_m+\alpha G_m]F_{n_0}, \end{aligned}$$

then both equations in (2.34) are satisfied. Using the fact that

$$\begin{aligned} \rho F_{n_0-1}+(n_0+1)F_{n_0+1}=(\rho +\theta +n_0)F_{n_0}, \end{aligned}$$

and $B-A=C\, G_{n_0}/F_{n_0}$, we obtain from (2.36)

2.40 $$\begin{aligned} C\left( -\dfrac{G_{n_0}}{F_{n_0}}F_{n_0+1}+G_{n_0+1}\right) =-\dfrac{1}{n_0+1}. \end{aligned}$$

Then using the Wronskian identity in (2.32), with $n=n_0$, and (2.38) we see that

2.41 $$\begin{aligned} \alpha a H_m= \dfrac{n_0!\Gamma (\theta )e^{-\rho }}{\rho ^{n_0+\theta }}. \end{aligned}$$

Using the fact that $H_n$ satisfies (2.10) with $n=m$, (2.35) is equivalent to $B F_{m-1}+C G_{m-1}=D H_{m-1}$ and using (2.38) and (2.39) leads to

$$\begin{aligned} H_m\, F_{m-1}+\alpha H_m\, G_{m-1}= F_m\, H_{m-1}+\alpha G_m\, H_{m-1}, \end{aligned}$$

and thus

2.42 $$\begin{aligned} \alpha =\dfrac{F_m\, H_{m-1}-H_m\, F_{m-1}}{H_m\, G_{m-1}-G_m\, H_{m-1}}. \end{aligned}$$

Using (2.41) and (2.42) in (2.37)–(2.39), and then in (2.32) we have thus solved for $\widehat{P}_n(\theta )$, which we summarize below.

#### **Theorem 1**

For initial conditions $0\leqslant n_0\leqslant m$, the Laplace transform $\widehat{P}_n(\theta )=\int ^{\infty }_0e^{-\theta t}p_n(t)\, dt$ of the time dependent distribution of *N*(*t*) is given by

2.43 $$\begin{aligned} \widehat{P}_n(\theta )= & {} \dfrac{n_0!}{m!}\rho ^{m-n_0-1}\dfrac{F_{n_0}(\theta )\, H_n(\theta ;m)}{F_m(\theta )\ H_{m-1}(\theta ;m)-H_m(\theta ;m)\, F_{m-1}(\theta )},\quad n\geqslant m;\end{aligned}$$

2.44 $$\begin{aligned} \widehat{P}_n(\theta )= & {} \dfrac{n_0!\Gamma (\theta )e^{-\rho }}{\rho ^{n_0+\theta }}F_{n_0}(\theta )\nonumber \\&\times \left[ G_n(\theta )+\dfrac{H_m(\theta ;m)\, G_{m-1}(\theta )-G_m(\theta )\,H_{m-1}(\theta ;m)}{F_m(\theta )\, H_{m-1}(\theta ;m)-H_m(\theta ;m)\, F_{m-1}(\theta )}F_n(\theta )\right] ,\quad n_0\leqslant n\leqslant m;\nonumber \\ \end{aligned}$$

2.45 $$\begin{aligned} \widehat{P}_n(\theta )= & {} \dfrac{n_0!\Gamma (\theta )e^{-\rho }}{\rho ^{n_0+\theta }}F_n(\theta )\nonumber \\&\times \left[ G_{n_0}(\theta )+\dfrac{H_m(\theta ;m)\, G_{m-1}(\theta )-G_m(\theta )\,H_{m-1}(\theta ;m)}{F_m(\theta )\, H_{m-1}(\theta ;m)-H_m(\theta ;m)\, F_{m-1}(\theta )}F_{n_0}(\theta )\right] ,\quad 0\leqslant n\leqslant n_0.\nonumber \\ \end{aligned}$$

Here $$F_n$$$$G_n$$, and $$H_n$$ are given by the contour integrals in (2.13), (2.15) and (2.22).

Thus far we have established this result only for $0<n_0<m$. However, it holds also if $n_0=0$. We need only verify that (2.44) satisfies the boundary equation (2.8), which becomes $\widehat{P}_1(\theta )-(\rho +\theta )\widehat{P}_0(\theta )=-1$ if $n_0=0$. But $G_1-(\rho +\theta )G_0$ can be computed from (2.15) as

2.46 $$\begin{aligned} G_1-(\rho +\theta )G_0&-\dfrac{1}{2\pi i}\int _{C_1}\dfrac{e^{\rho z}}{z^2(z-1)^{\theta }}[1-(\rho +\theta )z]\, dz\nonumber \\&=\dfrac{1}{2\pi i}\int _{C_1}\left\{ -\rho \dfrac{e^{\rho z}}{(z-1)^{\theta }}+\dfrac{d}{dz}\left[ \dfrac{e^{\rho z}}{z(z-1)^{\theta -1}}\right] \right\} \, dz\nonumber \\&=-\dfrac{e^{\rho }\rho ^{\theta }}{\Gamma (\theta )}. \end{aligned}$$

Since $F_1=(\rho +\theta )F_0$, using (2.46) and (2.44) with $n_0=0$ yields

$$\begin{aligned} \widehat{P}_1(\theta )-(\rho +\theta )\widehat{P}_0(\theta )=-1. \end{aligned}$$

We can also show that if $n_0=m$, the expressions in Theorem 1 satisfy $(m+\eta )\widehat{P}_{m+1}+\rho \widehat{P}_{m-1}-(\rho +\theta +m)\widehat{P}_m=-1$, corresponding to initial conditions $N(0)=m$, i.e., starting with all *m* servers occupied but no one in the queue.

We note that if $n_0=m$, (2.43)–(2.45) somewhat simplify, to

2.47 $$\begin{aligned} \widehat{P}_n(\theta )=\dfrac{\rho ^{-1}}{F_m\, H_{m-1}-H_m\,F_{m-1}}{\left\{ \begin{array}{ll} F_m\, H_n,&{}n\geqslant m\\ H_m\, F_n,&{}0\leqslant n\leqslant m. \end{array}\right. } \end{aligned}$$

Next we assume that $N(0)=n_0>m$. Now we must solve the homogeneous form of (2.9) with the boundary condition in (2.8), and these imply that $\widehat{P}_n(\theta )$ must be proportional to $F_n$ for all $0\leqslant n\leqslant m$. Thus now $G_n$ will not enter the analysis. For *n* large, $\widehat{P}_n(\theta )$ must again be proportional to $H_n$, which has the appropriate decay as $n\rightarrow \infty$. Now we write

2.48 $$\begin{aligned} \widehat{P}_n(\theta )={\left\{ \begin{array}{ll} \widetilde{A} F_n(\theta ),&{}0\leqslant n\leqslant m\\ \widetilde{B} H_n(\theta ;m)+ \widetilde{C} I_n(\theta ;m),&{}m\leqslant n\leqslant n_0\\ \widetilde{D}\, H_n(\theta ;m),&{}n\geqslant n_0. \end{array}\right. } \end{aligned}$$

Imposing the continuity conditions at $n=n_0$ and $n=m$ yields

2.49 $$\begin{aligned} \widetilde{A} F_m= & {} \widetilde{B} H_m+\widetilde{C} I_m\end{aligned}$$

2.50 $$\begin{aligned} \widetilde{D} H_{n_0}= & {} \widetilde{B} H_{n_0}+\widetilde{C} I_{n_0}. \end{aligned}$$

Setting $n=m$ in (2.10) then yields

2.51 $$\begin{aligned}&(m+\eta )\left[ \widetilde{B} H_{m+1}+\widetilde{C} I_{m+1}\right] +\rho \widetilde{A} F_{m-1}\nonumber \\&\quad =[\rho +\theta +m+(n-m)\eta ]\left[ \widetilde{B} H_m+\widetilde{C} I_m\right] \end{aligned}$$

and (2.10) with $n=n_0$ and the right side replaced by $-\delta (n,n_0)=-1$ leads to

2.52 $$\begin{aligned}&[m+(n_0-m+1)\eta ]\widetilde{D} H_{n_0+1}+\rho (\widetilde{B} H_{n_0+1}+\widetilde{C} I_{n_0-1})\nonumber \\&\quad -[\rho +\theta +m+(n_0-m)\eta ]\widetilde{D} H_{n_0}=-1. \end{aligned}$$

Thus (2.49)–(2.52) yields four equations for the four unknowns $\widetilde{A}$, $\widetilde{B}$, $\widetilde{C}$, $\widetilde{D}$. They can be solved similarly to (2.37)–(2.39), and we give below only the final result.

#### **Theorem 2**

For initial conditions $n_0\geqslant m$, $\widehat{P}_n(\theta )$ is given by

2.53 $$\begin{aligned} \widehat{P}_n(\theta )= & {} \dfrac{1}{\rho }e^{-\rho /\eta }\left( \dfrac{\eta }{\rho }\right) ^{n_0-m-1+\frac{\theta +m}{\eta }}\Gamma \left( \dfrac{\theta }{\eta }\right) \Gamma \left( n_0-m+1+\dfrac{m}{\eta }\right) \nonumber \\&\times \left[ I_{n_0}+\dfrac{I_m\, F_{m-1}-I_{m-1}\, F_m}{F_m\, H_{m-1}-H_m\, F_{m-1}}\, H_{n_0}\right] \, H_n,\quad n\geqslant n_0;\end{aligned}$$

2.54 $$\begin{aligned} \widehat{P}_n(\theta )= & {} \dfrac{1}{\rho }e^{-\rho /\eta }\left( \dfrac{\eta }{\rho }\right) ^{n_0-m-1+\frac{\theta +m}{\eta }}\Gamma \left( \dfrac{\theta }{\eta }\right) \Gamma \left( n_0-m+1+\dfrac{m}{\eta }\right) \nonumber \\&\times \left[ I_{n}+\dfrac{I_m\, F_{m-1}-I_{m-1}\, F_m}{F_m\, H_{m-1}-H_m\, F_{m-1}}\, H_{n}\right] \, H_{n_0},\quad m\leqslant n\leqslant n_0;\end{aligned}$$

2.55 $$\begin{aligned} \widehat{P}_n(\theta )= & {} \dfrac{1}{\rho }\left( \dfrac{\rho }{\eta }\right) ^{m-n_o}\dfrac{\Gamma \left( n_0-m+1+\frac{m}{\eta }\right) }{\Gamma \left( 1+\frac{m}{\eta }\right) }\dfrac{H_{n_0}\, F_n}{F_m\, H_{m-1}-H_m\, F_{m-1}},\quad 0\leqslant n\leqslant m.\nonumber \\ \end{aligned}$$

Here $F_n$, $H_n$ and $I_n$ are given by the contour integrals in (2.11), (2.22) and (2.24).

We note that if $n_0=m$, expression (2.54) is not needed, and then (2.53) and (2.55) agree with the expression(s) in (2.47). Setting $n_0=m$ in (2.53) and using

2.56 $$\begin{aligned} I_m\, H_{m-1}-I_{m-1}\, H_m=\dfrac{e^{\rho /\eta }}{\Gamma \left( \frac{\theta }{\eta }\right) }\dfrac{1}{\Gamma \left( \frac{m}{\eta }+1\right) }\left( \dfrac{\rho }{\eta }\right) ^{\frac{\theta +m}{\eta }-1}, \end{aligned}$$

which follows from (2.31), we obtain (2.47) for $n\geqslant m$.

### Limiting case: steady state

We proceed to examine some limiting cases of Theorems 1 and 2, where the expressions simplify, sometimes considerably. First we consider the steady state limit of $p_n(t)$ as $t\rightarrow \infty$, which corresponds to the limit of $\theta \widehat{P}_n(\theta )$ as $\theta \rightarrow 0$. First, observe that as $\theta \rightarrow 0$, (2.11) and (2.15) yields

2.57 $$\begin{aligned} F_n(0)=\dfrac{\rho ^n}{n!}=G_n(0), \end{aligned}$$

while (2.22) and (2.24) lead to

2.58 $$\begin{aligned} H_n(0;m)&=\left( \dfrac{\rho }{\eta }\right) ^{n-m+m/\eta }\dfrac{1}{\Gamma \left( n-m+1+\frac{m}{\eta }\right) }=I_n(0;m). \end{aligned}$$

Now consider $n_0\geqslant m$, where Theorem 2 applies. At $\theta =0$,

2.59 $$\begin{aligned} F_m(0)\, H_{m-1}(0;m)=F_{m-1}(0)\, H_m(0;m) \end{aligned}$$

and $I_m(0;m)\, F_{m-1}(0)=I_{m-1}(0;m)\, F_m(0)$, in view of (2.57) and (2.58). To estimate the various terms in (2.53)–(2.55) as $\theta \rightarrow 0$, we first compute

2.60 $$\begin{aligned} \Delta _1\equiv {}&\dfrac{d}{d\theta }[F_m\, H_{m-1} -H_m\, F_{m-1}]\Big \vert _{\theta =0}\nonumber \\ ={}&F'_m(0)\, H_{m-1}(0;m)+F_m(0)\, H'_{m-1}(0;m)\nonumber \\&-H'_m(0;m)\, F_{m-1}(0)-H_m(0;m)\, F'_{m-1}(0). \end{aligned}$$

Using (2.57) and (2.22) we have

2.61 $$\begin{aligned} F_m&(0)\, H'_{m-1}(0;m)-F_{m-1}(0)\, H'_m(0;m)\nonumber \\&=\dfrac{\rho ^{m-1}}{m!}\left[ \rho H'_{m-1}(0;m)-mH'_m(0;m)\right] \nonumber \\&=\dfrac{\rho ^{m-1}}{m!}\dfrac{1}{2\pi i}\int _{C_1}\left[ \dfrac{-\log (z-1)}{\eta }\dfrac{\rho e^{\rho z/\eta }}{z^{m/\eta }}+\dfrac{m}{\eta }\dfrac{\log (z-1)}{z^{1+m/\eta }}e^{\rho z/\eta }\right] \, dz\nonumber \\&=\dfrac{\rho ^{m-1}}{m!}\dfrac{1}{2\pi i}\int _{C_1}\log (z-1)\, \dfrac{d}{dz}\left[ -\dfrac{e^{\rho z/\eta }}{z^{m/\eta }}\right] \, dz\nonumber \\&=\dfrac{\rho ^{m-1}}{m!}\dfrac{1}{2\pi i}\int _{C_1}\dfrac{e^{\rho z/\eta }}{z^{m/\eta }}\dfrac{1}{z-1}\, dz\nonumber \\&=\dfrac{\rho ^{m-1}}{m!}\sum ^{\infty }_{\ell =0}\left[ \dfrac{1}{2\pi i}\int _{C_1}\dfrac{e^{\rho z/\eta }}{z^{\ell +1+m/\eta }}\, dz\right] \nonumber \\&=\dfrac{\rho ^{m-1}}{m!}\sum ^{\infty }_{\ell =0}\left( \dfrac{\rho }{\eta }\right) ^{\ell +\frac{m}{\eta }}\dfrac{1}{\Gamma \left( \ell +1+\frac{m}{\eta }\right) }. \end{aligned}$$

We can take $|z|>1$ on $C_1$, and then expand $(z-1)^{-1}$ as a Laurent series on $C_1$. Using (2.58) and (2.11) we have

2.62 $$\begin{aligned} H_{m-1}(0;m)\,&F'_m(0)-H_m(0;m)\, F'_{m-1}(0)\nonumber \\&=\dfrac{H_m(0;m)}{\rho }\left[ mF'_m(0)-\rho F'_{m-1}(0)\right] \nonumber \\&=\dfrac{H_m(0)}{\rho }\dfrac{1}{2\pi i}\int _{C_0}-\log (1-z)\left[ \dfrac{me^{\rho z}}{z^{m+1}}-\dfrac{\rho e^{\rho z}}{z^m}\right] \, dz\nonumber \\&=\dfrac{H_m(0)}{\rho }\dfrac{1}{2\pi i}\int _{C_0}\log (1-z)\, \dfrac{d}{dz}\left( \dfrac{e^{\rho z}}{z^m}\right) \, dz\nonumber \\&=\dfrac{H_m(0)}{\rho }\dfrac{1}{2\pi i}\int _{C_0}\dfrac{e^{\rho z}}{1-z}\dfrac{1}{z^m}\, dz\nonumber \\&=\rho ^{-1}\left( \dfrac{\rho }{\eta }\right) ^{m/\eta }\dfrac{1}{\Gamma \left( 1+\frac{m}{\eta }\right) }\sum ^{m-1}_{J=0}\dfrac{\rho ^J}{J!}, \end{aligned}$$

where now on $C_0$ we can expand $(1-z)^{-1}$ as $\sum ^{\infty }_{\ell =0}z^{\ell }$, since $|z|<1$. Combining (2.61) with (2.62), (2.60) then yields

2.63 $$\begin{aligned} \Delta _1=\left( \dfrac{\rho }{\eta }\right) ^{m/\eta }\dfrac{1}{\rho }\left[ \dfrac{1}{\Gamma \left( 1+\frac{m}{\eta }\right) }\sum ^{m-1}_{J=0}\dfrac{\rho ^J}{J!}+\dfrac{\rho ^m}{m!}\sum ^{\infty }_{\ell =0}\dfrac{(\rho /\eta )^{\ell }}{\Gamma \left( \ell +1+\frac{m}{\eta }\right) }\right] . \end{aligned}$$

Now let $\Delta _2=\frac{d}{d\theta }[F_m\, I_{m-1}-I_m\, F_{m-1}\Big \vert _{\theta =0}$. Since $I_m=H_m$ when $\theta =0$, the difference between $\Delta _1$ and $\Delta _2$ is

2.64 $$\begin{aligned} \Delta _1-\Delta _2&=F_m(0)\left[ H'_{m-1}(0;m)-I'_{m-1}(0;m)\right] \nonumber \\&\quad - F_{m-1}(0)\left[ H'_m(0;m)-I'_m(0;m)\right] \nonumber \\&=\dfrac{\rho ^{m-1}}{m!}\dfrac{1}{2\pi i}\left( \int _{C_1}-\int _{C_2}\right) \left( \dfrac{1}{z-1}\dfrac{e^{\rho z/\eta }}{z^{m/\eta }}\right) \, dz, \end{aligned}$$

when we used (2.24) and calculations similar to those in (2.61). But the difference between the contour integrals over $C_1$ and over $C_2$ is simply the residue from the pole at $z=-1$, and thus

2.65 $$\begin{aligned} \Delta _1-\Delta _2=\dfrac{\rho ^{m-1}}{m!}e^{\rho /\eta }. \end{aligned}$$

Using (2.63)–(2.65) we thus have

2.66 $$\begin{aligned} \lim _{\theta \rightarrow 0}\left\{ \left[ I_{n_0}+\dfrac{I_m\, F_{m-1}-I_{m-1}\, F_m}{F_m\, H_{m-1}-H_m\, F_{m-1}}\, H_{n_0}\right] H_n\right\} =H_{n_0}(0;m)\, H_n(0;m)\left[ 1-\dfrac{\Delta _2}{\Delta _1}\right] , \end{aligned}$$

and (2.66) can be used in view of (2.53) and (2.54), for both $n\in [m,n_0]$ and $n\geqslant n_0$. Then $\theta \Gamma (\theta /\eta )\rightarrow \eta$ as $\theta \rightarrow 0$ and $F_m\, H_{m-1}-H_m\, F_{m-1}=\theta \Delta _1+O(\theta ^2)$. We have thus obtained the steady state limit from Theorem 2 as stated below (see e.g., Garnett et al. 2002; Ward 2012).

#### **Corollary 1**

The steady state distribution is

2.67 $$\begin{aligned} p_n(\infty )&=K\dfrac{\rho ^m}{m!}\left( \dfrac{\rho }{\eta }\right) ^{n-m}\dfrac{\Gamma \left( 1+\frac{m}{\eta }\right) }{\Gamma \left( n-m+1+\frac{m}{\eta }\right) },\quad n\geqslant m,\end{aligned}$$

2.68 $$\begin{aligned} p_n(\infty )&=K\dfrac{\rho ^n}{n!},\quad 0\leqslant n\leqslant m, \end{aligned}$$

with

2.69 $$\begin{aligned} K=\left[ \sum ^{m-1}_{J=0}\dfrac{\rho ^J}{J!}+\dfrac{\rho ^m}{m!}\sum ^{\infty }_{\ell =0}\left( \dfrac{\rho }{\eta }\right) ^{\ell }\dfrac{\Gamma \left( 1+\frac{m}{\eta }\right) }{\Gamma \left( \ell +1+\frac{m}{\eta }\right) }\right] ^{-1}. \end{aligned}$$

Note that *K* and $$\Delta _1$$ are related by $$\rho \Delta _1\Gamma (1+m/\eta )(\rho /\eta )^{-m/\eta }$$$$K=1$$. While we obtained Corollary 1 from Theorem 2, which applies for $$n_0\geqslant m$$, the result is independent of $$n_0$$ and Corollary 1 will also follow from Theorem 1 using very similar calculations to those in (2.60)–(2.66), which we omit. Of course, $$p_n(\infty )$$ is more easily obtained by letting $$t\rightarrow \infty $$ in (2.2)–(2.5) and solving the resulting elementary difference equations.

### Limiting cases: extreme abandonments rates

Next we evaluate Theorems 1 and 2 for the special cases $\eta =1$, $\eta \rightarrow 0^+$ (vanishing abandonment effects) and $\eta \rightarrow \infty$. For $\eta =1$ the model reduces to the standard infinite server $M/M/\infty$ queue, and from Theorems 1 and 2 we obtain the following.

#### **Corollary 2**

When $\eta =1$ the Laplace transform of $p_n(t)$ is given by

2.70 $$\begin{aligned} \widehat{P}_n(\theta )=\dfrac{\Gamma (\theta )n!e^{-\rho }}{\rho ^{n_0+\theta }}{\left\{ \begin{array}{ll} F_{n_0}(\theta )\, G_n(\theta ),&{}n\geqslant n_0\\ G_{n_0}(\theta )\, F_n(\theta ),&{}0\leqslant n\leqslant n_0. \end{array}\right. } \end{aligned}$$

A spectral representation of $p_n(t)$ is then

2.71 $$\begin{aligned} p_n(t)=\dfrac{n_0!e^{-\rho }}{\rho ^{n_0}}\sum ^{\infty }_{k=0}\dfrac{\rho ^k}{k!}e^{-kt}F_{n_0}(-k)\, F_n(-k) \end{aligned}$$

where

2.72 $$\begin{aligned} F_n(-k)&=\dfrac{1}{2\pi i}\int _{C_0}\dfrac{(1-z)^k}{z^{n+1}}e^{\rho z}\, dz =\sum ^{\min \{k,n\}}_{j=0}\left( \begin{matrix} k\\ j \end{matrix}\right) (-1)^j\dfrac{\rho ^{n-j}}{(n-j)!}, \end{aligned}$$

and an alternate form is given by

2.73 $$\begin{aligned} p_n(t)&=\rho ^n\left( 1-e^{-t}\right) ^n\exp \left[ -\rho \left( 1-e^{-t}\right) \right] \nonumber \\&\quad \times \sum ^{\min \{n,n_0\}}_{j=0}\left( \begin{matrix} n_0\\ j \end{matrix} \right) \rho ^{-j}e^{-jt}\left( 1-e^{-t}\right) ^{n_0-2j}\dfrac{1}{(n-j)!}, \end{aligned}$$

and then $$p_n(\infty )=e^{-\rho }\rho ^n/n!$$.

We have already seen that when $\eta =1$, $H_n=G_n$ and $I_n=F_n$ and we have the Wronskian identities in (2.31) and (2.32). Then both Theorems 1 and 2 reduce to (2.70), and we need not distinguish the cases $n_0\gtrless m$, as *m* disappears altogether from the expressions. Now, from (2.11) and (2.15) it is clear that $F_n(\theta )$ and $G_n(\theta )$ are entire functions of $\theta$, for every *n*. Thus the only singularities of  (2.70) are the poles of $\Gamma (\theta )$, which occur at $\theta =-N$, $N=0,1,2,\ldots$ and the corresponding residues are $(-1)^N/N!$. When $\theta =-N$, $G_n$ and $F_n$ are no longer linearly independent, and in fact $G_n(-N)=(-1)^NF_n(-N)$, which follows by comparing (2.15) with (2.11). Thus evaluating the contour integral $p_n(t)=(2\pi i)^{-1} \int _{\text {Br}} e^{\theta t}\widehat{P}_n(\theta )\, d\theta$ (where ${{\mathrm{\hbox {Re}}}}(\theta )>0$ on the vertical Bromwich contour) as a residue series we obtain precisely (2.71), with (2.72). To obtain the expression in (2.73) we represent the $F_n(-k)$ in (2.71) as contour integrals, yielding

2.74 $$\begin{aligned} p_n(t)&=\dfrac{n_0!}{\rho ^{n_0}}e^{-\rho \left( 1-e^{-t}\right) }\dfrac{1}{(2\pi i)^2}\int _{C_0}\int _{C_0}\dfrac{\exp \left( \rho zwe^{-t}\right) }{z^{n_0+1}w^{n+1}}\nonumber \\&\quad \times \exp \left[ \rho \left( 1-e^{-t}\right) z+\rho \left( 1-e^{-t}\right) w\right] \, dz\, dw\nonumber \\&=e^{-\rho \left( 1-e^{-t}\right) }\dfrac{1}{2\pi i}\int _{C_0}\dfrac{e^{\rho \left( 1-e^{-t}\right) w}}{w^{n+1}}\left[ we^{-t}+1-e^{-t}\right] ^{n_0}\, dw. \end{aligned}$$

Then expanding $$\big [we^{-t}+1-e^{-t}\big ]^{\!n_0}$$ using the binomial theorem leads to (2.73). Note that as $$t\rightarrow 0$$$$\left( 1-e^{-t}\right) ^{n+n_0-2j}\rightarrow 0$$ unless $$n=n_0$$ and $$j=n$$, so that $$p_n(0)=\delta (n,n_0)$$. As $$t\rightarrow \infty $$ only the term with $$j=0$$ in (2.73) remains and we obtain the steady state Poisson distribution. If $$\eta =1$$ it is easier to solve (2.2)–(2.6) using the generating function $$\mathcal {G}(t,u)=\sum ^{\infty }_{n=0}p_n(t)u^n$$ which leads to the first order PDE

2.75 $$\begin{aligned} \dfrac{\partial \mathcal {G}}{\partial t}+(u-1)\dfrac{\partial \mathcal {G}}{\partial u}=\rho (u-1)\mathcal {G},\quad \mathcal {G}(0,u)=u^{n_0}, \end{aligned}$$

whose solution is

2.76 $$\begin{aligned} \mathcal {G}(t,u)=\exp \left[ \rho \left( 1-e^{-t}\right) (u-1)\right] \left[ 1+(u-1)e^{-t}\right] ^{n_0}. \end{aligned}$$

Inverting the generating function then regains (2.73).

Next we let $\eta \rightarrow 0^+$, so that the model reduces to the *m*-server *M* / *M* / *m* queue. Then we obtain the following.

#### **Corollary 3**

When $\eta =0$ the Laplace transform of $p_n(t)$ is given by, for $0\leqslant n_0\leqslant m$,

2.77 $$\begin{aligned} \widehat{P}_n(\theta )&=\dfrac{n_0!}{m!}\rho ^{m-n_0}\dfrac{F_{n_0}(\theta )[A(\theta )]^{n-m}}{(m+1)F_{m+1}(\theta )-A(\theta )mF_m(\theta )},\quad n\geqslant m,\end{aligned}$$

2.78 $$\begin{aligned} A(\theta )&=\dfrac{1}{2m}\left[ m+\rho +\theta -\sqrt{(m+\rho +\theta )^2-4m\rho }\right] ,\end{aligned}$$

2.79 $$\begin{aligned} \widehat{P}_n(\theta )&=\dfrac{n_0!\Gamma (\theta )e^{-\rho }}{\rho ^{n_0+\theta }}\left[ G_n+\dfrac{mA\,G_m-(m+1)G_{m+1}}{(m+1)F_{m+1}-mA\,F_m}F_n\right] F_{n_0},\quad n_0\leqslant n\leqslant m,\end{aligned}$$

2.80 $$\begin{aligned} \widehat{P}_n(\theta )&=\dfrac{n_0!\Gamma (\theta )e^{-\rho }}{\rho ^{n_0+\theta }}\left[ G_{n_0}+\dfrac{mA\,G_m-(m+1)G_{m+1}}{(m+1)F_{m+1}-mA\,F_m}F_{n_0}\right] F_n,\quad 0\leqslant n\leqslant n_0. \end{aligned}$$

For $n_0\geqslant m$ we have

2.81 $$\begin{aligned} \widehat{P}_n(\theta )&=B^{m-n_0}\dfrac{F_n}{(m+1)F_{m+1}-Am\, F_m},\quad 0\leqslant n\leqslant m,\end{aligned}$$

2.82 $$\begin{aligned} B(\theta )&=\dfrac{1}{2m}\left[ \rho +m+\theta +\sqrt{(m+\rho +\theta )^2-4m\rho }\right] ,\end{aligned}$$

2.83 $$\begin{aligned} \widehat{P}_n(\theta )&=\dfrac{1}{\sqrt{(m+\rho +\theta )^2-4m\rho }}\nonumber \\&\times \left[ B^{n-n_0} +A^{n-m}\, B^{m-n_0}\dfrac{(m+1)F_{m+1}-Bm\, F_m}{Am\,F_m-(m+1)F_{m+1}}\right] ,\quad m\leqslant n\leqslant n_0,\end{aligned}$$

2.84 $$\begin{aligned} \widehat{P}_n(\theta )&=\dfrac{1}{\sqrt{(m+\rho +\theta )^2-4m\rho }}\nonumber \\&\times \left[ A^{n-n_0} +B^{m-n_0}\, A^{n-m}\dfrac{(m+1)F_{m+1}-Bm\, F_m}{Am\, F_m-(m+1)F_{m+1}}\right] ,\quad n\geqslant n_0. \end{aligned}$$

We also note that the transient distribution for the *M* / *M* / *m* model was previously obtained, in different forms, by Saaty (1960) and van Doorn (1979). In van Doorn (1979) spectral methods are used, while in Saaty (1960) the Laplace transform is expressed in terms of hypergeometric functions.

To establish (2.77)–(2.84) we need to evaluate $H_n(\theta ;m)$ and $I_n(\theta ;m)$ for $\eta \rightarrow 0^+$. We write $H_n$ in (2.22) as

2.85 $$\begin{aligned} H_n=\dfrac{1}{2\pi i}\int _{C_1}\dfrac{1}{z^{n+1-m}}\exp \left[ \dfrac{1}{\eta }f(\theta ,z)\right] \, dz \end{aligned}$$

where

2.86 $$\begin{aligned} f(\theta ,z)=\rho z-m\log z-\theta \log (z-1), \end{aligned}$$

so that the integrand has saddle points where $\partial f/\partial z=0$, and this occurs at

2.87 $$\begin{aligned} z=Z_{\pm }(\theta )\equiv \dfrac{1}{2\rho }\left[ \rho +\theta +m\pm \sqrt{(\rho +\theta +m)^2-4\rho m}\right] . \end{aligned}$$

We can take $|z|>1$ on $C_1$ and then the saddle at $Z_+$ determines the asymptotic behavior of $H_n$ as

2.88 $$\begin{aligned} H_n\sim & {} \sqrt{\dfrac{\eta }{2\pi }}Z_+^{m-n-1}\left[ \dfrac{m}{Z^2_+}+\dfrac{\theta }{(Z_+-1)^2}\right] ^{-1/2}\nonumber \\&\times \, \exp \left\{ \dfrac{1}{\eta }\left[ \rho Z_+-m\log Z_+-\theta \log (Z_+-1)\right] \right\} ,\quad \eta \rightarrow 0^+. \end{aligned}$$

It follows that $H_{n-1}/H_n\sim Z_+$ in this limit,

2.89 $$\begin{aligned} \dfrac{H_m\, G_{m-1}-G_m\, H_{m-1}}{F_m\, H_{m-1}-H_m\, F_{m-1}}\rightarrow \dfrac{G_{m-1}-Z_+\, G_m}{F_m\, F_+-F_{m-1}},\quad \eta \rightarrow 0^+, \end{aligned}$$

and

2.90 $$\begin{aligned} \dfrac{H_n}{F_m\, H_{m-1}-H_m\, F_{m-1}}\rightarrow \dfrac{Z_+^{m-n}}{F_m\, Z_+-F_{m-1}},\quad n\rightarrow 0^+. \end{aligned}$$

But, $\rho F_{m-1}+(m+1)F_{m+1}=(m+\rho +\theta )F_m$ so that

$$\begin{aligned} \rho Z_+F_m-\rho F_{m-1}&=\rho F_m(Z_+-1)-(m+\theta )F_m+(m+1)F_{m+1}\\&=(m+1)F_{m+1}-Am\, F_m \end{aligned}$$

as $A=1/Z_+$ and $Z_{\pm }$ satisfy the quadratic equation

$$\begin{aligned} \rho Z_{\pm }^2-(\rho +m+\theta )Z_{\pm }+m=0. \end{aligned}$$

Hence (2.43) reduces to (2.77) as $\eta \rightarrow 0^+$. Also, (2.79) and (2.80) follow from (2.44) and (2.45), in view of (2.89) and the fact that

$$\begin{aligned} \dfrac{G_{m-1}-G_m\, Z_+}{F_m\, Z_+-F_{m-1}}\cdot \dfrac{\rho }{\rho } = \dfrac{Am\, G_m-(m+1)G_{m+1}}{(m+1)F_{m+1}-Am\, f_m}. \end{aligned}$$

Now consider $n_0\geqslant m$. We shall obtain (2.81)–(2.84) from Theorem 2. We must then expand $I_n(\theta ;m)$ for $\eta \rightarrow 0^+$. Using the saddle point method we find that

2.91 $$\begin{aligned} I_n\sim & {} \sqrt{\dfrac{\eta }{2\pi }}Z_-^{m-n-1}\left[ \dfrac{m}{Z_-^2}+\dfrac{\theta }{(1-Z_-)^2}\right] ^{-1/2}\nonumber \\&\times \,\exp \left\{ \dfrac{1}{\eta }\left[ \rho Z_--m\log Z_--\theta \log (1-Z_-)\right] \right\} , \end{aligned}$$

as the expansion of (2.24), which involves the contour $C_2$, is determined by the other saddle point in (2.87). Using (2.88) with *n* replaced by $n_0$, (2.91), and Stirling’s formula we obtain, after a lengthy calculation, the following limit (as $\eta \rightarrow 0^+$):

2.92 $$\begin{aligned}&I_n(\theta ;m)H_{n_0}(\theta ;m)\, \Gamma \left( \dfrac{\theta }{\eta }\right) \, \Gamma \left( n_0+1-m+\dfrac{m}{\eta }\right) \nonumber \\&\quad \times \, \dfrac{1}{\rho }e^{-\rho /\eta }\left( \dfrac{\eta }{\rho }\right) ^{n_0-m-1+\frac{\theta +m}{\eta }}\rightarrow Z_-^{n_0-n}\dfrac{1}{\sqrt{(\rho +\theta +m)^2-4m\rho }}. \end{aligned}$$

After factoring out $I_n$, the bracketed factor in (2.54) becomes

2.93 $$\begin{aligned}&1+\dfrac{H_n}{I_n} \dfrac{I_m\, F_{m-1}-I_{m-1}\, F_m}{F_m\, H_{m-1}-H_m\, F_{m-1}}\nonumber \\&\qquad \rightarrow 1+ \dfrac{Z_-^{n-m}\, F_{m-1}-Z_-^{n+1-m}\, F_m}{Z_+^{n+1-m}\, F_m-Z_+^{n-m}\, F_{m-1}}\nonumber \\&\qquad =1+Z_+^{m-n}Z_-^{n-m}\dfrac{F_{m-1}-Z_-\, F_m}{Z_+\, F_m-F_{m-1}}\nonumber \\&\qquad =1+A^{n-m}\, B^{m-n}\dfrac{(m+1)F_{m+1}-Bm\, F_m}{Am\, F_m-(m+1)F_{m+1}}, \end{aligned}$$

where we again used $A Z_+=1$, $B Z_-=1$ and the quadratic equation satisfied by $Z_{\pm }$. With (2.92) and (2.93) the expression in (2.54) becomes that in (2.83). A completely analogous calculation shows that (2.53) leads to (2.84) as $\eta \rightarrow 0^+$. Now consider (2.55). As $\eta \rightarrow 0^+$, by Stirling’s formula,

$$\begin{aligned} \left( \dfrac{\rho }{\eta }\right) ^{m-n_0}\dfrac{\Gamma \left( n_0+1-m+\frac{m}{\eta }\right) }{\Gamma \left( 1+\frac{m}{\eta }\right) }\rightarrow m^{n_0-m}\rho ^{m-n_0} \end{aligned}$$

and we also use (2.90) with *n* replaced by $n_0$, and

$$\begin{aligned} \rho [F_m\, Z_+-F_{m-1}]=(m+1)F_{m+1}-Am\, F_m. \end{aligned}$$

Then (2.55) goes to the limit in (2.81), since $\rho Z_+/m=B$. This completes the proof of Corollary 3.

In the limit $\eta \rightarrow \infty$, we expect our results to reduce to the Erlang loss model, or the *M* / *M* / *m* / *m* queue. We then obtain the following.

#### **Corollary 4**

As $\eta \rightarrow \infty$ the Laplace transform of $p_n(t)$, for $0\leqslant n_0\leqslant m$, approaches the limit

2.94 $$\begin{aligned} \widehat{P}_n(\theta )&=\dfrac{n_0!\Gamma (\theta )e^{-\rho }}{\rho ^{n_0+\theta }}\, F_{n_0}[G_n+\omega F_n],\quad n_0\leqslant n\leqslant m\end{aligned}$$

2.95 $$\begin{aligned} \widehat{P}_n(\theta )&=\dfrac{n_0!\Gamma (\theta )e^{-\rho }}{\rho ^{n_0+\theta }}\, F_n[G_{n_0}+\omega F_{n_0}],\quad 0\leqslant n\leqslant n_0, \end{aligned}$$

where

$$\begin{aligned} \omega =\dfrac{(m+1)G_{m-1}-\rho G_m}{\rho F_m-(m+1)F_{m+1}}. \end{aligned}$$

In particular the blocking probability $p_m(t)$ has the Laplace transform

2.96 $$\begin{aligned} \widehat{P}_m(\theta )=\dfrac{n_0!}{m!}\rho ^{m-n_0}\dfrac{F_{n_0}(\theta )}{(m+1)F_{m+1}(\theta )-\rho F_m(\theta )}. \end{aligned}$$

Note that (2.35) follows by setting $n=m$ in (2.94) and using the Wronskian $\widetilde{W}_m$ in (2.32). To establish Corollary 4, we note that by expanding the integrand in (2.22) for $\eta \rightarrow \infty$ we obtain

2.97 $$\begin{aligned} H_n=\delta (n,m) +\dfrac{1}{\eta }\Big [\rho \delta (n,m+1) -\dfrac{1}{2\pi i}\int _{C_1}\dfrac{m\log z+\theta \log (z-1)}{z^{n+1-m}}\, dz\Big ]+O(\eta ^{-2}) \end{aligned}$$

and thus $H_m(\theta ;m)=1+O(\eta ^{-1})$ and $\eta H_{m+1}(\theta ;m) \rightarrow \rho$ as $\eta \rightarrow \infty$. Since

$$\begin{aligned} \rho H_{m-1}+(m+\eta )H_{m+1} =(\rho +m+\theta )H_m \end{aligned}$$

we have $H_{m-1}\rightarrow (\theta +m)/\rho$ as $\eta \rightarrow \infty$. Thus, as $\eta \rightarrow \infty$,

2.98 $$\begin{aligned} \dfrac{H_m\, G_{m-1}-G_m\, H_{m-1}}{F_m\, H_{m-1}-H_m\, F_{m-1}}\rightarrow \dfrac{\rho G_{m-1}-(\theta +m)G_m}{(\theta +m)F_m-\rho F_{m-1}}. \end{aligned}$$

But

$$\begin{aligned} (m+1)G_{m+1}-\rho G_m=-\rho G_{m-1}+(\theta +m)G_m \end{aligned}$$

and

$$\begin{aligned} (m+1)F_{m+1}-\rho F_m=-\rho F_{m-1}+(\theta +m)F_m, \end{aligned}$$

so with (2.98), (2.47) and (2.45) yields (2.94) and(2.95) in the limit $\eta \rightarrow \infty$.

The blocking probability in (2.96) may also be written as

2.99 $$\begin{aligned} \widehat{P}_m(\theta )=\dfrac{n_0!}{m!}\rho ^{m-n_0}\dfrac{F_{n_0}(\theta )}{\theta F_m(\theta +1)}, \end{aligned}$$

since $\theta F_m(\theta +1)=(m+1)F_{m+1}(\theta )-\rho F_m(\theta )$, which follows from (2.11) with $n=m$ and an integration by parts. If $N(0)=0$ (starting with an empty system) we obtain from (2.99)

2.100 $$\begin{aligned} \widehat{P}_m(\theta )=\dfrac{\Gamma (\theta )}{\sum \nolimits ^m_{\ell =0}\left( \begin{matrix} m\\ \ell \end{matrix}\right) \rho ^{-\ell }\Gamma (\theta +\ell +1)}. \end{aligned}$$

Previously expressions for the Laplace transform of the blocking probability were obtained by Jagerman (2000), who showed that (if $n_0=0$)

2.101 $$\begin{aligned} \widehat{P}_m(\theta )=\dfrac{\Gamma (\theta )}{\int \limits _0^{\infty }e^{-\xi }\xi ^{\theta }\left( 1+\frac{\xi }{m\rho }\right) ^m\, d\xi }, \end{aligned}$$

and this can easily be shown to agree with both (2.100) and (2.99), as

2.102 $$\begin{aligned} \dfrac{\rho ^m}{m!}\int ^{\infty }_0e^{-\xi }\xi ^{\theta }\left( 1+\dfrac{\xi }{m\rho }\right) ^m\, d\xi = \dfrac{\Gamma (\theta +1)}{2\pi i}\int _{C_0}\dfrac{e^{\rho z}}{z^{m+1}}(1-z)^{-\theta -1}\, dz \end{aligned}$$

follows by expanding both integrands using the binomial theorem.

For general $n_0\in [0,m]$ the blocking probability is given by

2.103 $$\begin{aligned} \widehat{P}_m(\theta )=\dfrac{ \sum ^{n_0}_{\ell =0}\left( \begin{matrix} n_0\\ \ell \end{matrix}\right) \rho ^{-\ell }\,\Gamma (\theta +\ell )}{\sum ^m_{\ell =0}\left( \begin{matrix} m\\ \ell \end{matrix}\right) \rho ^{-\ell }\,\Gamma (\theta +\ell +1)}. \end{aligned}$$

Since the expressions in Theorems 1 and 2 and even Corollaries 3 and 4, are quite complicated, it is useful to expand these in various asymptotic limits. One such limit would have $m\rightarrow \infty$, $\rho \rightarrow \infty$ with $m/\rho \rightarrow 1$ and $m-\rho =O(\sqrt{m})$. This is a diffusion limit, sometimes referred to as the Halfin–Whitt regime. Here we would scale *n*, $n_0$ and $\rho$, for $m\rightarrow \infty$, as

2.104 $$\begin{aligned} \rho =m-\sqrt{m}\beta ,\quad n=m+\sqrt{m}x,\quad n_0=m+\sqrt{m}x_0, \end{aligned}$$

and *x*, $x_0$ and $\beta$ are *O*(1). In this limit we can approximate the contour integrals $F_n$, $G_n$, $H_n$ and $I_n$ by simpler special functions, namely parabolic cylinder functions. We discuss this limit in detail in van Leeuwaarden and Knessl (2011) for the *M* / *M* / *m* model with $\eta =0$, and in Leeuwaarden and Knessl (2012) for the $M/M/m+M$ model with $\eta >0$. We can obtain then $p_n(t)\sim m^{-1/2}P(x,t)$ where *P* will satisfy a parabolic PDE, which we explicitly solved in van Leeuwaarden and Knessl (2011), Leeuwaarden and Knessl (2012). An alternate approach is to evaluate Theorems 1 and 2, or Corollary 3 in the limit in (2.104), and thus identify *P*(*x*, *t*) directly. We shall discuss in more detail the limit in (2.104) for the first passage distributions. We also comment that the transient behavior of the *M* / *M* / *m* / *m* model was analyzed thoroughly in Knessl (1990) and Xie and Knessl (1993), for $m\rightarrow \infty$ and various cases of $\rho$, including the scaling in (2.104). There we used mostly singular perturbation methods, but equivalent results could be obtained using Corollary 4 and methods for asymptotically expanding integrals.

## First passage times

### Main result: Laplace transform of the first passage time

Here we compute the distribution of the time for the number *N*(*t*) of customers to reach some level $n_*$, which may be viewed as a measure of congestion. We take $n_*>m$, for otherwise the problem reduces to that of the $M/M/\infty$ or *M* / *M* / *m* / *m* models. Thus we define the stopping time

3.1 $$\begin{aligned} \tau (n_*)=\min \{t:N(t)=n_*\}, \end{aligned}$$

and its conditional distribution is

3.2 $$\begin{aligned} Q_n(t) dt={{\mathrm{\hbox {Prob}}}}\left[ \tau (n_*)\in (t,t+dt)\mid N(0)=n\right] . \end{aligned}$$

When $n=n_*$ we clearly have

3.3 $$\begin{aligned} Q_{n_*}(t)=\delta (t) \end{aligned}$$

and for $n<n_*$, $Q_n(t)$ satisfies the backward Kolmogorov equation(s)

3.4 $$\begin{aligned} Q'_0(t)= & {} \rho Q_1(t)-\rho Q_0(t)\end{aligned}$$

3.5 $$\begin{aligned} Q'_n(t)= & {} \rho Q_{n+1}(t)+n Q_{n-1}(t)-(\rho +n)Q_n(t),\quad 1\leqslant n\leqslant m,\end{aligned}$$

3.6 $$\begin{aligned} Q'_n(t)= & {} \rho Q_{n+1}(t)+[m+(n-m)\eta ] Q_{n-1}(t)\nonumber \\&-\,[\rho +m+(n-m)\eta ]Q_n(t),\quad m\leqslant n\leqslant n_*. \end{aligned}$$

To analyze (3.3)–(3.6) we first introduce the Laplace transform

$$\begin{aligned} \widehat{Q}_n(\theta )=\int _0^{\infty }e^{-\theta t}Q_n(t)\, dt \end{aligned}$$

and, expecting that $Q_n(0)=0$ for $n<n_*$, we obtain

3.7 $$\begin{aligned}&\widehat{Q}_{n_*}(\theta )=1\end{aligned}$$

3.8 $$\begin{aligned}&\rho \widehat{Q}_1(\theta )=(\rho +\theta ) \widehat{Q}_0(\theta )\end{aligned}$$

3.9 $$\begin{aligned}&(\rho +n+\theta ) \widehat{Q}_n(\theta )=\rho \widehat{Q}_{n+1}(\theta )+n\widehat{Q}_{n-1}(\theta ),\quad 1\leqslant n\leqslant m,\end{aligned}$$

3.10 $$\begin{aligned}&[\rho +m+(n-m)\eta +\theta ]\widehat{Q}_n (\theta ) =\rho \widehat{Q}_{n+1}(\theta )\nonumber \\&\quad +\,[m+(n-m)\eta ]\widehat{Q}_{n-1}(\theta ),\quad m\leqslant n\leqslant n_*-1. \end{aligned}$$

The recurrences in (3.9) and (3.10) are similar to those in (2.9) and (2.10), and indeed we can convert the former to the latter by setting

3.11 $$\begin{aligned} \widehat{Q}_n(\theta )&=\rho ^{-n}\dfrac{n!}{m!}R_n(\theta ),\quad 0\leqslant n\leqslant m\end{aligned}$$

3.12 $$\begin{aligned} \widehat{Q}_n(\theta )&=\rho ^{-n}\eta ^{n-m}\dfrac{\Gamma \left( n-m+1+\frac{m}{\eta }\right) }{\Gamma \left( 1+\frac{m}{\eta }\right) }R_n(\theta ),\quad m\leqslant n\leqslant n_*. \end{aligned}$$

Then from (3.7) and (3.12) we have

3.13 $$\begin{aligned} R_{n_*}(\theta )=\eta ^{m-n_*}\rho ^{n_*}\dfrac{\Gamma \left( 1+\frac{m}{\eta }\right) }{\Gamma \left( n_*-m+1+\frac{m}{\eta }\right) }, \end{aligned}$$

and $R_n(\theta )$ will satisfy

$$\begin{aligned} (\rho +n+\theta )R_n=(n+1)R_{n+1}+\rho R_{n-1} \end{aligned}$$

for $0<n<m$, which is just the homogeneous version of (2.9), while for $n>m$, $R_n(\theta )$ will satisfy (2.10). Also, $R_1(\theta )=(\rho +\theta )R_0(\theta )$, so that $R_n(\theta )$ will satisfy the boundary equation in (2.8). We can thus write $R_n$ in terms of the special functions $F_n$, $G_n$, $H_n$, $I_n$ that we introduced in Sect.  2, and since $F_n$ satisfies (2.8) we write

3.14 $$\begin{aligned} R_n(\theta )=c_1F_n(\theta ),\quad 0\leqslant n\leqslant m \end{aligned}$$

and

3.15 $$\begin{aligned} R_n(\theta )=c_2H_n(\theta ;m)+c_3I_n(\theta ;m),\quad m\leqslant n\leqslant n_*. \end{aligned}$$

In view of (3.15) and (3.13) we have

3.16 $$\begin{aligned} c_2H_{n_*}+c_3I_{n_*}=\eta ^{m-n_*}\rho ^{n_*}\dfrac{\Gamma \left( 1+\frac{m}{\eta }\right) }{\Gamma \left( n_*-m+1+\frac{m}{\eta }\right) } \end{aligned}$$

and if both (3.14) and (3.15) apply for $n=m$ we have the continuity equation

3.17 $$\begin{aligned} c_1F_m=c_2H_m+c_3I_m. \end{aligned}$$

Finally, using (3.5) with $n=m$ and noting that, in view of (3.11) and (3.12),

3.18 $$\begin{aligned} \begin{aligned} \widehat{Q}_m-\widehat{Q}_{m-1}&=\rho ^{-m}\left[ R_m-\dfrac{\rho }{m}R_{m-1}\right] ,\\ \widehat{Q}_{m+1}-\widehat{Q}_{m}&=\rho ^{-m-1}\left[ (m+\eta )R_{m-1}-\rho R_{m}\right] , \end{aligned} \end{aligned}$$

we find that $(m+\eta )R_{m+1}+\rho R_{m-1}=(\theta +\rho +m)R_m$ and thus

3.19 $$\begin{aligned} (m+\eta )\left[ c_2H_{m+1}+c_3I_{m+1}\right] +\rho c_1 F_{m-1} =(\theta +\rho +m)c_1F_m. \end{aligned}$$

Then (3.16), (3.17) and (3.19) yield three equations for the unknowns $c_1$, $c_2$, $c_3$. After some algebra and use of (2.31) with $n=m$ we obtain $R_n$, and then $\widehat{Q}_n$ follows from (3.11) and (3.12). We summarize below the final results.

#### **Theorem 3**

The distribution of the first passage time to a level $n_*(>m)$ has the Laplace transform $\widehat{Q}_n(\theta )=E\left[ e^{-\theta \tau (n_*)}\mid N(0)=n\right]$:

3.20 $$\begin{aligned} \widehat{Q}_n(\theta )= & {} \rho ^{n_*-n}\dfrac{n!}{m!}\eta ^{m-n_*+1}\left( \dfrac{\rho }{\eta }\right) ^{\frac{m+\theta }{\eta }}\dfrac{e^{\rho /\eta }}{\Gamma \left( \frac{\theta }{\eta }\right) \Gamma \left( n_*-m+1+\frac{m}{\eta }\right) }\nonumber \\&\times \dfrac{F_n}{(m+\eta )(H_{n_*}\, I_{m+1}-I_{n_*}\, H_{m+1})F_m+(m+1)(H_m\, I_{n_*}-H_{n_*}\, I_m)F_{m+1}},\nonumber \\&0\leqslant n\leqslant m,\end{aligned}$$

3.21 $$\begin{aligned} \widehat{Q}_n(\theta )= & {} \rho ^{n_*-n}\eta ^{n-n_*}\dfrac{\Gamma \left( n-m+1+\frac{m}{\eta }\right) }{\Gamma \left( n_*-m+1+\frac{m}{\eta }\right) }\nonumber \\&\times \dfrac{(m+\eta )(H_n\,I_{m+1}-I_n\, H_{m+1})F_m+(m+1)(H_m\, I_n-H_n\, I_m)F_{m+1}}{(m+\eta )(H_{n_*}\,I_{m+1}-I_{n_*}\, H_{m+1})F_m+(m+1)(H_m\, I_{n_*}-H_{n_*}\, I_m)F_{m+1}},\nonumber \\&m\leqslant n\leqslant n_*. \end{aligned}$$

Note that actually (3.20) can be used even if $n=m+1$ and it then agrees with (3.21). Similarly, (3.21) holds even if $n=m-1$. If $\eta =1$ we have $F_n=I_n$ and then both (3.20) and (3.21) reduce to

3.22 $$\begin{aligned} \widehat{Q}_n(\theta )=\dfrac{n!}{n_*!}\rho ^{n_*-n}\dfrac{F_n(\theta )}{F_{n_*}(\theta )},\quad 0\leqslant n\leqslant n_* \end{aligned}$$

which is the result for the $M/M/\infty$ model. We can again get results for the standard *M* / *M* / *m* model by letting $\eta \rightarrow 0^+$ in Theorem 3. Using the asymptotic results in (2.88) and (2.91), after some calculations that we omit we obtain the following.

#### **Corollary 5**

For the *M* / *M* / *m* model the first passage distribution to a level $n_*(>m)$ is given by

3.23 $$\begin{aligned} \widehat{Q}_n(\theta )= & {} \rho ^{m-n}\dfrac{n!}{m!}\sqrt{(\theta +m+\rho )^2-4m\rho }\nonumber \\&\times \dfrac{F_n(\theta )}{\rho F_m(Z_+Z_-^{n_*-m} - Z_-Z_+^{n_*-m})+(m+1)F_{m+1}(Z_+^{n_*-m}-Z_-^{n_*-m})},\nonumber \\&0\leqslant n\leqslant m \end{aligned}$$

and

3.24 $$\begin{aligned} \widehat{Q}_n(\theta )= & {} \dfrac{\rho F_m(Z_+Z_-^{n-m}-Z_-Z_+^{n-m})+(m+1)F_{m+1}(Z_+^{n-m}-Z_-^{n-m})}{\rho F_m(Z_+Z_-^{n_*-m}-Z_-Z_+^{n_*-m})+(m+1)F_{m+1}(Z_+^{n_*-m}-Z_-^{n_*-m})},\nonumber \\&m\leqslant n\leqslant n_*. \end{aligned}$$

Here $Z_{\pm }$ are as in (2.87).

Using the fact that $F_n(0)=\rho ^{n}/n!$ and $Z_{\pm }(0)=[m+\rho \pm |m-\rho |]/(2\rho )$ we can easily verify that $\widehat{Q}_n(0)=1$ for all *n*, so that the density is properly normalized. We shall discuss later the mean first passage time, which is equal to $-\widehat{Q}'_n(0)$.

### Halfin–Whitt regime

We next consider the limit in (2.104) in Corollary 5, also scaling the exit point $n_*$ as

3.25 $$\begin{aligned} n_*= m+\sqrt{m}b,\quad 0<b<\infty . \end{aligned}$$

From (2.87) we obtain, using (2.104),

$$\begin{aligned} Z_{\pm }=1+\dfrac{1}{2\sqrt{m}}\left[ \beta \pm \sqrt{\beta ^2+4\theta }\right] +O(m^{-1}),\quad m\rightarrow \infty \end{aligned}$$

and hence

3.26 $$\begin{aligned} Z_{\pm }^{n-m}\sim \exp \left[ \dfrac{1}{2}\left( \beta \pm \sqrt{\beta ^2+4\theta }\right) x\right] . \end{aligned}$$

By scaling $z=1-\xi /\sqrt{m}$ in (2.11) and noting that $\rho z-n\log z=\rho +(x+\beta )\xi +\frac{1}{2}\xi ^2+o(1)$ with the Halfin–Whitt scaling in (2.104), the integral in (2.11) can be approximated by

3.27 $$\begin{aligned} F_n(\theta )\sim&\dfrac{1}{2\pi i}\dfrac{m^{\theta /2}e^{\rho }}{\sqrt{m}}\int _{\text {Br}_+}\xi ^{-\theta }e^{(x+\beta )\xi }e^{\xi ^2/2}\, d\xi \nonumber \\&=\dfrac{m^{\theta /2}e^{\rho }}{\sqrt{2\pi m}}e^{-(x+\beta )^2/4}D_{-\theta }(-x-\beta ), \end{aligned}$$

where $D_p(z)$ is the parabolic cylinder function of index *p* and argument  *z*. In (3.27) the approximating contour $\text {Br}_+$ is a vertical contour in the $\xi$-plane, on which ${{\mathrm{\hbox {Re}}}}(\xi )>0$, and $\xi ^{-\theta }$ is defined to be analytic for ${{\mathrm{\hbox {Re}}}}(\xi )>0$ and real and positive for $\xi$ real and positive. In view of (2.104), setting $n=m$ corresponds to $x=0$ and thus

3.28 $$\begin{aligned} F_m(\theta )\sim \dfrac{m^{\theta /2}e^{\rho }}{\sqrt{2\pi m}}e^{-\beta ^2/4}D_{-\theta }(-\beta ),\quad m\rightarrow \infty . \end{aligned}$$

A similar calculation shows that

3.29 $$\begin{aligned} F_{m+1}(\theta )-F_m(\theta )\sim \dfrac{m^{\theta /2}e^{\rho }}{\sqrt{2\pi } m}e^{-\beta ^2/4}D_{1-\theta }(-\beta ),\quad m\rightarrow \infty \end{aligned}$$

and we note that the difference $F_{m+1}-F_m$ is smaller than $F_m$ by a factor of $m^{-1/2}$.

We write the denominator in (3.23) and (3.24) as

3.30 $$\begin{aligned}&\rho F_m\left[ Z_+Z_-^{n_*-m}-Z_-Z_+^{n_*-m}\right] -(m+1)F_{m+1}\left[ Z_-^{n_*-m}-Z_+^{n_*-m}\right] \nonumber \\&\quad =-(m+1)(F_{m+1}-F_m)\left( Z_-^{n_*-m}-Z_+^{n_*-m}\right) \nonumber \\&\qquad +Z_{-}^{n_*-m}F_m(\rho Z_+-m-1)+Z_+^{n_*-m}F_m(-\rho Z_-+m+1)\nonumber \\&\quad \sim \dfrac{m^{\theta /2}}{\sqrt{2\pi }} e^{-\beta ^2/4}e^{b\beta /2}\bigg \{2D_{1-\theta }(-\beta )\sinh \left( \dfrac{b}{2}\sqrt{\beta ^2+4\theta }\right) \nonumber \\&\qquad +e^{-\sqrt{\beta ^2\!{}+{}\!4\theta }b/2}\dfrac{1}{2}\left[ -\beta \!{}+{}\!\sqrt{\beta ^2+4\theta }\right] D_{-\theta }(-\beta )\nonumber \\&\qquad +e^{\sqrt{\beta ^2\!{}+{}\!4\theta }b/2}\dfrac{1}{2}\left[ \beta +\sqrt{\beta ^2\!{}+{}\!4\theta }\right] D_{-\theta }(-\beta )\bigg \}. \end{aligned}$$

Here we used (3.28), (3.29), (3.26), and also

$$\begin{aligned} \rho Z_{\pm }-m-1\sim \dfrac{1}{2}\sqrt{m}\left[ -\beta \pm \sqrt{\beta ^2+4\theta }\right] . \end{aligned}$$

The expansion of the numerator in  (3.24) follows by replacing *b* by *x* in (3.30). In the limit in (2.104) we also have, using Stirling’s formula,

3.31 $$\begin{aligned} \rho ^{m-n}\dfrac{n!}{m!}\sqrt{(\theta +m+\rho )^2-4m\rho }\sim e^{x\beta }e^{x^2/2}\sqrt{m}\sqrt{\beta ^2+4\theta }. \end{aligned}$$

We summarize below our final results.

#### **Corollary 6**

In the limit $m\rightarrow \infty$, with the scaling in (2.104) and (3.25), the transform of the first passage distribution $\widehat{Q}_n(\theta )$ for the *M* / *M* / *m* model has the limit $\widehat{\mathcal {P}}(x,\theta )$ where

3.32 $$\begin{aligned} \widehat{\mathcal {P}}(x,\theta )= e^{x\beta /2}e^{x^2/4}\sqrt{\beta ^2+4\theta } e^{-\beta b/2}\dfrac{D_{-\theta }(-\beta -x)}{\Lambda (\theta ;b,\beta )},\quad -\infty <x\leqslant 0 \end{aligned}$$

with

3.33 $$\begin{aligned} \Lambda (\theta ;b,\beta )={}&\sqrt{\beta ^2+4\theta } \cosh \left( \dfrac{b}{2}\sqrt{\beta ^2+4\theta }\right) D_{-\theta }(-\beta )\nonumber \\&+\sinh \left( \dfrac{b}{2}\sqrt{\beta ^2+4\theta }\right) \left[ 2D_{1-\theta }(-\beta )+\beta D_{-\theta }(-\beta )\right] \end{aligned}$$

and

3.34 $$\begin{aligned} \widehat{\mathcal {P}}(x,\theta )=\dfrac{\Lambda (\theta ;x,\beta )}{\Lambda (\theta ;b,\beta )},\quad 0\leqslant x\leqslant b. \end{aligned}$$

We have previously obtained these results in Fralix et al. (2014), by directly solving the parabolic PDE satisfied by the diffusion approximation. Since $2D_{1-\theta }(-\beta )+\beta D_{-\theta }(-\beta )=-2D'_{-\theta }(-\beta )$, Corollary 6 agrees with Theorems 1 and 2 in Fralix et al. (2014).

Now, we can also consider the Halfin–Whitt limit for the first passage distribution in the $M/M/m+M$ model (with a fixed $\eta >0$), and then Theorem 3 reduces to the following.

#### **Corollary 7**

For $m\rightarrow \infty$ with the scaling in  (2.104) and (3.25), $\widehat{Q}(\theta )$ in the $M/M/m+M$ model has the limit $\widehat{\mathcal {P}}(x,\theta )$ where

3.35 $$\begin{aligned} \widehat{\mathcal {P}}(x,\theta )= & {} \dfrac{e^{\beta (x-b)/2}e^{(x^2-\eta b^2)/4}\sqrt{2\pi }D_{-\theta }(-\beta -x)}{\Gamma \left( \frac{\theta }{\eta }\right) \left[ D_{-\theta /\eta }\left( \frac{\beta +\eta b}{\sqrt{\eta }}\right) \Lambda _1+D_{-\theta /\eta }\left( \frac{-\beta -\eta b}{\sqrt{\eta }}\right) \Lambda _2\right] },\quad -\infty <x\leqslant 0,\nonumber \\ \end{aligned}$$

3.36 $$\begin{aligned} \Lambda _1= & {} -\sqrt{\eta }D'_{-\theta /\eta }\left( \dfrac{-\beta }{\sqrt{\eta }}\right) D_{-\theta }(-\beta )+D_{-\theta /\eta }\left( \dfrac{-\beta }{\sqrt{\eta }}\right) D'_{-\theta }(-\beta ),\end{aligned}$$

3.37 $$\begin{aligned} \Lambda _2= & {} -\sqrt{\eta }D'_{-\theta /\eta }\left( \dfrac{\beta }{\sqrt{\eta }}\right) D_{-\theta }(-\beta )-D_{-\theta /\eta }\left( \dfrac{\beta }{\sqrt{\eta }}\right) D'_{-\theta }(-\beta ),\end{aligned}$$

3.38 $$\begin{aligned} \widehat{\mathcal {P}}(x,\theta )= & {} e^{\beta (x-b)/2}e^{\eta \left( x^2-b^2\right) /4}\nonumber \\&\times \dfrac{D_{-\theta /\eta }\left( \dfrac{\beta +\eta x}{\sqrt{\eta }}\right) \Lambda _1+ D_{-\theta /\eta }\left( \dfrac{-\beta -\eta x}{\sqrt{\eta }}\right) \Lambda _2}{D_{-\theta /\eta }\left( \dfrac{\beta +\eta b}{\sqrt{\eta }}\right) \Lambda _1+D_{-\theta /\eta }\left( \dfrac{-\beta -\eta b}{\sqrt{\eta }}\right) \Lambda _2},\quad 0\leqslant x<b. \end{aligned}$$

We can show that as $\eta \rightarrow 0^+$, Corollary 7 reduces to Corollary 6, so that the order of the limits of small $\eta$ and that in (2.104) may be, in this case, interchanged. While we can obtain Corollary 7 from Theorem 3 by expanding $H_n$ and $I_n$ in the limit in (2.104), where

$$\begin{aligned} H_n\sim \sqrt{\dfrac{\eta }{2\pi m}} e^{\rho /\eta }\left( \dfrac{m}{\eta }\right) ^{\frac{\theta }{2\eta }}e^{-(\eta x+\beta )^2/(4\eta )} D_{-\theta /\eta }\left( \dfrac{\eta x+\beta }{\sqrt{\eta }}\right) , \end{aligned}$$

and a similar expression holds for $I_n$, it is easier to simply obtain a limiting PDE from (3.5) and (3.6) (or limiting ODE from (3.9) and (3.10)) and solve it. If $\sqrt{m}\widehat{Q}_n(\theta )\rightarrow \widehat{\mathcal {P}} (x,\theta )$ then $\widehat{\mathcal {P}}$ must satisfy

3.39 $$\begin{aligned} \theta \widehat{\mathcal {P}}= & {} \widehat{\mathcal {P}}_{xx}-(\beta +\eta x)\widehat{\mathcal {P}}_x,\quad x<0,\end{aligned}$$

3.40 $$\begin{aligned} \theta \widehat{\mathcal {P}}= & {} \widehat{\mathcal {P}}_{xx}-(\beta +x)\widehat{\mathcal {P}}_x,\quad 0<x<b, \end{aligned}$$

and the boundary condition is $\widehat{\mathcal {P}}(b,\theta )=1$. We also have the interface conditions $\widehat{\mathcal {P}}(0^-,\theta ) =\widehat{\mathcal {P}}(0^+,\theta )$ and $\widehat{\mathcal {P}}_x(0^-,\theta ) =\widehat{\mathcal {P}}_x(0^+,\theta )$, where subscripts denote partial derivatives. Setting

3.41 $$\begin{aligned} \widehat{\mathcal {P}}(x,\theta )&=e^{x^2/4}e^{\beta x/2}\widetilde{\mathcal {P}}(x,\theta ),\quad x<0\end{aligned}$$

3.42 $$\begin{aligned} \widehat{\mathcal {P}}(x,\theta )&=e^{\eta x^2/4}e^{\beta x/2}\widetilde{\mathcal {P}}(x,\theta ),\quad 0<x<b \end{aligned}$$

we obtain from (3.39) and (3.40)

3.43 $$\begin{aligned}&\widetilde{\mathcal {P}}_{xx}+\left[ \dfrac{1}{2}-\theta -\dfrac{1}{4}(\beta +x)^2\right] \widetilde{\mathcal {P}}=0,\quad x<0\end{aligned}$$

3.44 $$\begin{aligned}&\widetilde{\mathcal {P}}_{xx}+\left[ \dfrac{\eta }{2}-\theta -\dfrac{1}{4}(\beta +\eta x)^2\right] \widetilde{\mathcal {P}}=0,\quad 0<x<b, \end{aligned}$$

and $\widetilde{\mathcal {P}}$ and $\widetilde{\mathcal {P}}_x$ must also be continuous at $x=0$, in view of (3.41) and (3.42) and the continuity of $\widehat{\mathcal {P}}$ and $\widehat{\mathcal {P}}_x$. Also, the boundary condition is

3.45 $$\begin{aligned} \widetilde{\mathcal {P}}(b,\theta )=\exp \left[ -\dfrac{1}{4}\eta b^2-\dfrac{1}{2}\beta b\right] . \end{aligned}$$

Equation (3.43) is the parabolic cylinder equation of index $$-\theta $$, and its two linearly independent solution are $$D_{-\theta }(\beta +x)$$ and $$D_{-\theta }(-\beta -x)$$, for $$-\theta \ne 0,1,2,\dots $$. But as $$x\rightarrow -\infty $$$$D_{-\theta }(\beta +x)$$ has Gaussian growth in *x*, which would lead to $$\widehat{\mathcal {P}}$$ in (3.41) being roughly $$O(e^{x^2/2})$$ as $$x\rightarrow -\infty $$. Thus for $$x<0$$ the solution must be proportional to $$D_{-\theta }(-\beta -x)$$, hence we write

3.46 $$\begin{aligned} \widetilde{\mathcal {P}}(x,\theta )=a(\theta )D_{-\theta }(-\beta -x),\quad x<0. \end{aligned}$$

The equation in (3.44) may be transformed, by the substitution

$$\begin{aligned} y=(\beta +\eta x)/\sqrt{\eta }, \end{aligned}$$

into a parabolic cylinder equation of index $-\theta /\eta$, and thus for $x>0$ we have

3.47 $$\begin{aligned} \widetilde{\mathcal {P}}(x,\theta )=b(\theta )D_{-\theta /\eta }\left( \dfrac{\beta +\eta x}{\sqrt{\eta }}\right) +c(\theta )D_{-\theta /\eta }\left( \dfrac{-\beta -\eta x}{\sqrt{\eta }}\right) . \end{aligned}$$

The continuity conditions at $x=0$ then yield

3.48 $$\begin{aligned} a(\theta )D_{-\theta }(-\beta )=b(\theta )D_{-\theta /\eta }\left( \dfrac{\beta }{\sqrt{\eta }}\right) +c(\theta )D_{-\theta /\eta }\left( \dfrac{-\beta }{\sqrt{\eta }}\right) \end{aligned}$$

and

3.49 $$\begin{aligned} -a(\theta )D'_{-\theta }(-\beta )=b(\theta )\sqrt{\eta }D_{-\theta /\eta }\left( \dfrac{\beta }{\sqrt{\eta }}\right) -c(\theta )\sqrt{\eta }D'_{-\theta /\eta }\left( \dfrac{-\beta }{\sqrt{\eta }}\right) . \end{aligned}$$

Using the Wronskian identity

3.50 $$\begin{aligned} -D_{-\theta /\eta }\!\left( \dfrac{\beta }{\sqrt{\eta }}\right) \!D'_{-\theta /\eta }\!\left( \dfrac{-\beta }{\sqrt{\eta }}\right) \! \!{}-{}\!D_{-\theta /\eta }\!\left( \dfrac{-\beta }{\sqrt{\eta }}\right) \!D'_{-\theta /\eta }\!\left( \dfrac{\beta }{\sqrt{\eta }}\right) \!{} ={}\!\dfrac{\sqrt{2\pi }}{\Gamma \!\left( \frac{\theta }{\eta }\right) \!} \end{aligned}$$

we solve the system (3.45), (3.48) and (3.49), for the unknowns $a(\theta )$, $b(\theta )$, $c(\theta )$. We thus find that

3.51 $$\begin{aligned} b(\theta )=\dfrac{a(\theta )}{\sqrt{2\pi }}\Gamma \left( \dfrac{\theta }{\eta }\right) \Lambda _1,\quad c(\theta )=\dfrac{a(\theta )}{\sqrt{2\pi }}\Gamma \left( \dfrac{\theta }{\eta }\right) \Lambda _2, \end{aligned}$$

where the $\Lambda _j$ are as in (3.36) and (3.37), and

3.52 $$\begin{aligned} a(\theta )=\dfrac{\sqrt{2\pi }}{\Gamma \left( \frac{\theta }{\eta }\right) } \dfrac{e^{-\eta b^2/4}e^{-\beta b/2}}{D_{-\theta /\eta }\left( \frac{-\beta -\eta b}{\sqrt{\eta }}\right) \Lambda _2 +D_{-\theta /\eta }\left( \frac{\beta +\eta b}{\sqrt{\eta }}\right) \Lambda _1}. \end{aligned}$$

Using (3.51) and (3.52) in (3.46), (3.47), (3.41) and (3.42) gives the result in Corollary 7.

### Mean first passage time

Finally, we give below the mean first passage time,

3.53 $$\begin{aligned} q_n=E\left[ \tau (n_*)\mid N(0)=n\right] =\int ^{\infty }_0 tQ_n(t)\, dt=-\widehat{Q}'_n(0). \end{aligned}$$

#### **Corollary 8**

The conditional mean time to reach $N(t)=n_*$ starting from $N(0)=n\leqslant n_*$ is

3.54 $$\begin{aligned} q_n&=q_m+\sum ^{m-1}_{j=n}j!\rho ^{-j}\left[ \sum ^j_{\ell =0}\dfrac{\rho ^{\ell -1}}{\ell !}\right] ,\quad 0\leqslant n\leqslant m,\end{aligned}$$

3.55 $$\begin{aligned} q_m&=\dfrac{1}{\rho }\sum ^{n_*-1}_{J=m}\bigg [\left( \dfrac{\rho }{\eta }\right) ^{m-J}\dfrac{\Gamma \left( J-m+1+\frac{m}{\eta }\right) }{\Gamma \left( 1+\frac{m}{\eta }\right) }\sum ^m_{\ell =0}\dfrac{m!}{\ell !}\rho ^{\ell -m}\nonumber \\&\quad +\sum ^J_{\ell =m+1}\left( \dfrac{\rho }{\eta }\right) ^{\ell -J}\dfrac{\Gamma \left( J-m+1+\frac{m}{\eta }\right) }{\Gamma \left( \ell -m+1+\frac{m}{\eta }\right) }\bigg ],\end{aligned}$$

3.56 $$\begin{aligned} q_n&=\dfrac{1}{\rho }\sum ^{n_*-1}_{J=n}\sum ^J_{\ell =m+1}\left( \dfrac{\rho }{\eta }\right) ^{\ell -J}\dfrac{\Gamma \left( J-m+1+\frac{m}{\eta }\right) }{\Gamma \left( \ell -m+1+\frac{m}{\eta }\right) }\nonumber \\&\quad +\dfrac{1}{\rho }\left( \dfrac{\rho }{\eta }\right) ^{m}\dfrac{1}{\Gamma \left( 1+\frac{m}{\eta }\right) } \left[ \sum ^m_{\ell =0}\dfrac{m!}{\ell }\rho ^{\ell -m}\right] \nonumber \\&\quad \times \left[ \sum ^{n_*-1}_{J=n}\left( \dfrac{\eta }{\rho }\right) ^J\Gamma \left( J-m+1+\dfrac{m}{\eta }\right) \right] ,\quad m\leqslant n<n_*, \end{aligned}$$

with $$q_{n_*}=0$$.

We note that using (2.22) we have

3.57 $$\begin{aligned} H_n(0)=\left( \dfrac{\rho }{\eta }\right) ^{n-m+\frac{m}{\eta }}\dfrac{1}{\Gamma \left( n-m+1+\frac{m}{\eta }\right) } \end{aligned}$$

and the expression in (3.50) may also be written as

3.58 $$\begin{aligned} q_n=\dfrac{1}{\rho }\left[ \sum ^{n_*-1}_{J=n}\sum ^J_{\ell =m+1}\dfrac{H_{\ell }(0)}{H_J(0)}+ \sum ^{n_*-1}_{J=n}\dfrac{H_{m}(0)}{H_J(0)}\sum ^m_{\ell =0}\dfrac{m!}{\ell !}\rho ^{\ell -m}\right] ,\quad m\leqslant n<n_* \end{aligned}$$

By multiplying (3.4)–(3.6) by *t* and integrating from $t=0$ to $t=\infty$ we see that $q_n$ satisfies the recurrence(s)

3.59 $$\begin{aligned}&\rho (q_{n+1}-q_n)+n(q_{n-1}-q_n)=-1,\quad 0\leqslant n\leqslant m,\end{aligned}$$

3.60 $$\begin{aligned}&\rho (q_{n+1}-q_n)+[m+(n-m)\eta ](q_{n-1}-q_n)=-1,\quad m\leqslant n\leqslant n_*-1, \end{aligned}$$

with $q_{n_*}=0$. Solving the difference equations in (3.59) and (3.60) by elementary methods leads to Corollary 8. The same results can be obtained by computing $-\widehat{Q}'_n(0)$ using the expressions in Theorem 3.
